# *TaCYP81D5*, one member in a wheat cytochrome P450 gene cluster, confers salinity tolerance via reactive oxygen species scavenging

**DOI:** 10.1111/pbi.13247

**Published:** 2019-09-17

**Authors:** Meng Wang, Jiarui Yuan, Lumin Qin, Weiming Shi, Guangmin Xia, Shuwei Liu

**Affiliations:** ^1^ State Key Laboratory of Soil and Sustainable Agriculture Institute of Soil Science Chinese Academy of Sciences Nanjing China; ^2^ Key Laboratory of Plant Development and Environmental Adaptation Biology Ministry of Education School of Life Sciences Shandong University Qingdao China

**Keywords:** *Triticum aestivum*, *TaCYP81D5*, salinity tolerance, reactive oxygen species scavenging, *Zat12*

## Abstract

As one of the largest gene families in plants, the cytochrome P450 monooxygenase genes (*CYPs*) are involved in diverse biological processes including biotic and abiotic stress response. Moreover, P450 genes are prone to expanding due to gene tandem duplication during evolution, resulting in generations of novel alleles with the neo‐function or enhanced function. Here, the bread wheat (*Triticum aestivum*) gene *TaCYP81D5* was found to lie within a cluster of five tandemly arranged *CYP81D* genes, although only a single such gene (*BdCYP81D1*) was present in the equivalent genomic region of the wheat relative *Brachypodium distachyon*. The imposition of salinity stress could up‐regulate *TaCYP81D5,* but the effect was abolished in plants treated with an inhibitor of reactive oxygen species synthesis. In SR3, a wheat cultivar with an elevated ROS content, the higher expression and the rapider response to salinity of *TaCYP81D5* were related to the chromatin modification. Constitutively expressing *TaCYP81D5* enhanced the salinity tolerance both at seedling and reproductive stages of wheat via accelerating ROS scavenging. Moreover, an important component of ROS signal transduction, *Zat12*, was proven crucial in this process. Though knockout of solely *TaCYP81D5* showed no effect on salinity tolerance, knockdown of *BdCYP81D1* or all *TaCYP81D* members in the cluster caused the sensitivity to salt stress. Our results provide the direct evidence that *TaCYP81D5* confers salinity tolerance in bread wheat and this gene is prospective for crop improvement.

## Introduction

As one of the largest families of plant proteins, the cytochrome P450 monooxygenases (CYPs) are haem‐thiolate enzymes that are involved in various NADPH‐ and O_2_‐dependent hydroxylation reactions (Bak *et al*., 2011). In higher plants, CYPs, as versatile catalysts, play essential roles in the biosynthesis of considerable compounds and metabolites, such as antioxidants, phytohormones, structural polymers and signal molecules (Renault *et al*., 2014). The manipulation of *CYP* expression can result in alterations to both plant stature (Fernandez *et al*., 2009) and pathogen resistance (Koch *et al*., 2013). However, such attempt has been barely made for improving abiotic tolerance, particularly salinity tolerance, in crops.

The CYP81 subfamily includes multiple members, only few of which have been subjected to functional analysis. The *Medicago truncatula* CYP81Es act as isoflavone 2′‐ and 3′‐hydroxylases (Liu *et al*., 2003), while the *Sesamum indicum* SiCYP81Q1 protein catalyses the synthesis of the lignan sesamin (Ono *et al*., 2006). The *Arabidopsis thaliana* genome encodes 17 CYP81 proteins (nine CYP81Dx, four CYP81Fx, two CYP81Kx, one CYP81Gx and one CYP81Hx; Bak *et al*., 2011), among which only the function of CYP81Fs has been verified that it is critical in glucosinolate metabolism (Bednarek *et al*., 2009; Pfalz *et al*., 2011). While CYP81s clearly perform a variety of functions, almost all their encoding genes are inducible by abiotic stress, particularly by salt stress and oxidative stress (Liu *et al*., 2003; Narusaka *et al*., 2004; Shang *et al*., 2014). The activation of *AtCYP81D8* has been used as marker for the presence of reactive oxygen species (ROS; Baruah *et al*., 2009), although its *in planta* function is not clear. Accordingly, whether members of CYP81 subfamily do contribute to salinity tolerance and adaptations of other abiotic stresses needs further study.

Bread wheat (*Triticum aestivum*) is one of the major staple crops across the world and provides approximately 30% calories consumed by the world population. However, as a consequence of global climate change, seawater intrusion and urbanization, the soil salinity becomes quite severe which is a major constraint upon wheat grain yield (Munns and Gilliham, 2015). On the other hand, the complexity of bread wheat hexaploid genome greatly hampers the understanding of its genetic bases of salinity tolerance and hence its improvement via the genetic manipulation (Wang *et al*., 2018). Recent developments of whole genome sequencing have opened a door for wheat research (Wang *et al*., 2015b). Two major mechanisms underlying salinity tolerance, including leaf Na^+^ exclusion mediated by high‐affinity K^+^ transporters (HKTs) and ROS homoeostasis, have been addressed in wheat (Munns and Gilliham, 2015; Wang and Xia, 2018). Among them, the genetic framework of ROS homoeostasis was mostly depicted in a salinity‐tolerant bread wheat cultivar, Shanrong No. 3 (SR3). SR3 was generated via somatic hybridization between a salinity‐sensitive bread wheat cultivar Jinan177 (JN177) and tall wheatgrass (Liu *et al*., 2015). Physiologically, the stronger ROS accumulation is related to the higher salinity tolerance in SR3 against JN177 (Liu *et al*., 2014). Multiple genes regulating ROS production and/or scavenging, such as *TaSRO1* (Liu *et al*., 2014) and *TaOPR1* (Dong *et al*., 2013), are involved in this biochemical basis of SR3. Moreover, ‘genomic shock’ during the process of somatic hybridization leads to massive epigenetic variations, which is also associated with divergent expression patterns of salinity‐responsive genes between SR3 and JN177 (Wang *et al*., 2014). In animals, the status of DNA methylation is regulated by the level of ROS content (Franco *et al*., 2008). However, no direct evidence in whether the difference of ROS accumulation and ROS homeostasis maintenance between SR3 and JN177 affects DNA methylation, and its effect on gene expression, has been discovered.

In bread wheat, due to the lack of genome information in the past, only countable *CYP* genes have been identified and functional analysed (Ma *et al*., 2015; Nomura *et al*., 2005). Recently, genomic study has indicated that the nature of wheat genome, which contains high proportions of transposons and repetitive elements, makes it easily to generate duplicated gene fragments/alleles with non‐function, neo‐function or redundant function (Choulet *et al*., 2014; Wang *et al*., 2015b). Meanwhile, P450 genes have been found prone to expanding due to gene tandem duplication in plants (Nelson and Werck‐Reichhart, 2011; Yu *et al*., 2017). Here, *TaCYP81D5*, a wheat cytochrome P450 protein gene, was isolated from a salt stress‐related hotspot region which consisted of five tandemly distributed *CYP81Dx* genes. Functional analysis indicated *TaCYP81D5* contributes to salinity tolerance both at seedling and reproductive stages of bread wheat and this gene is prospective for crop improvement.

## Results

### The *TaCYP81Dx* gene family

A previous microarray analysis of the SR3 and JN177 transcriptomes (Liu *et al*., 2012) was able to show that the abundance of a probe (probe ID: ta_06616) encoding a family 81, subfamily D cytochrome P450, was raised by the imposition of salinity stress and was more abundant in SR3 than in JN177 (Figure S1a). One transcript, *TaCYP81D5*, covering this probe was discovered in our previously constructed cDNA libraries of SR3 and JN177 (Wang *et al*., 2015a). Intriguingly, this gene was located within a cluster of salinity‐responsive *TaCYP81Dx* genes (transcript IDs TraesCS5B01G402700, TraesCS5B01G402800, TraesCS5B01G402900, TraesCS5B01G403000 and TraesCS5B01G403100) mapping to the long arm of chromosome 5B (Zhang *et al*., 2016). Inspection of the v1.0 wheat reference genome (http://www.wheatgenome.org/) showed that the five *TaCYP81Dx* genes were tandemly arranged; namely, no other gene was present between any two of these five *TaCYP81Dx* genes.

**Figure 1:**
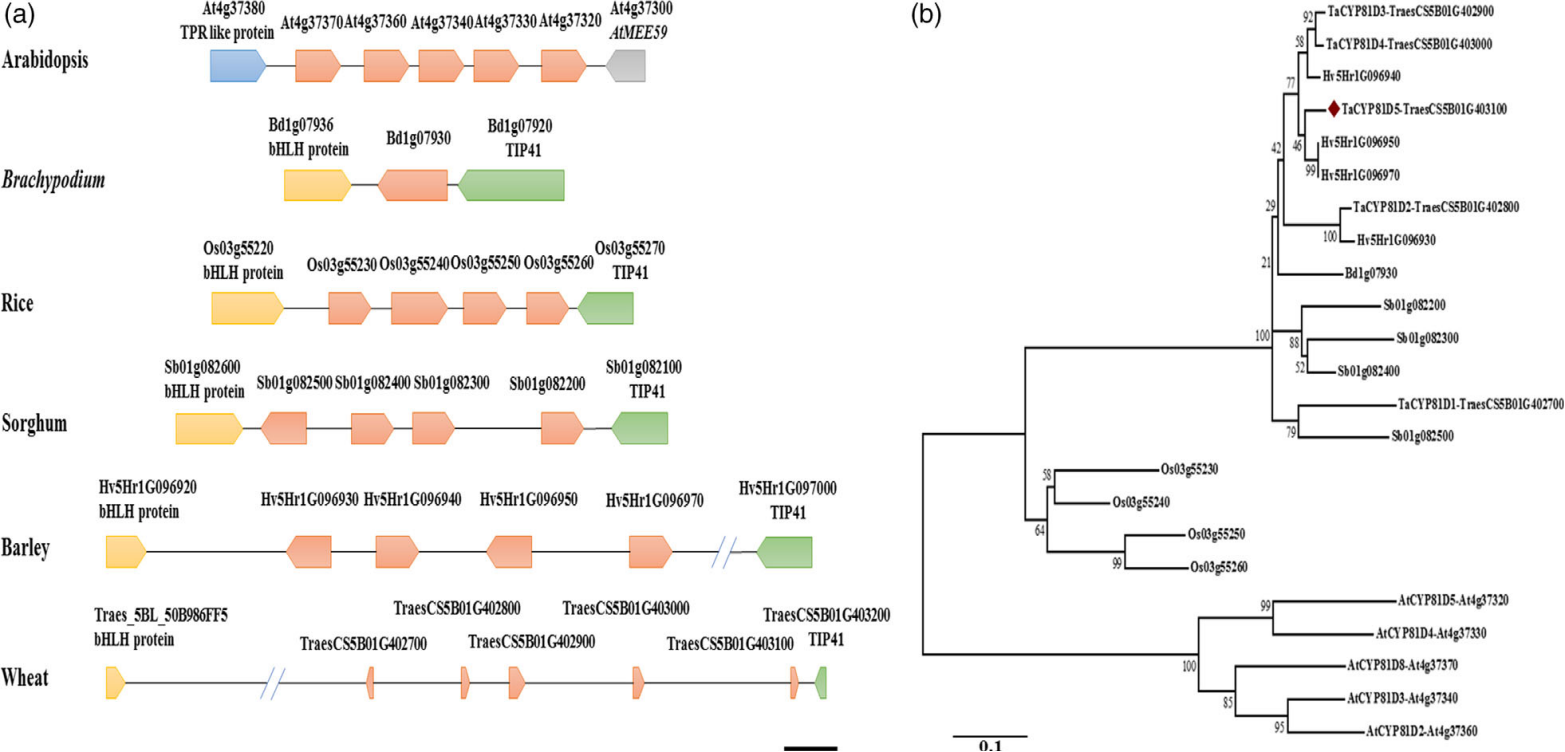
The genomic location and phylogeny of the family of *TaCYP81Dx* genes. (a) The genomic location of *CYP81Dx* homologs (marked by an orange pentagon) in *A*. *thaliana*,* B. distachyon*, rice, sorghum, barley and wheat. Bar: ~1500 nt in *A*. *thaliana*,* B. distachyon*, rice, sorghum and barley, but ~10 000 nt in wheat. (b) Phylogenetic analysis of *CYP81Dx* genes in *A*. *thaliana*,* B. distachyon*, rice, sorghum, barley and wheat. Scale of 0.1 corresponds to the number of amino acid substitutions per site.

There are five *AtCYP81Dx* genes present in *A. thaliana*, also arranged in tandem (Bak *et al*., 2011). However, the orientation style, the flanking genes and the genomic structure (two introns in *AtCYP81Dx* while only one in *TaCYP81Dx*) of this *AtCYP81Dx* cluster were different from those of *TaCYP81Dx* cluster (Figure 1a), likely owing to the divergent evolution between monocots and dicots. A scan of other grass species genomes revealed one homolog in *Brachypodium distachyon* and four in rice, sorghum and barley, in each case lying within a region flanked by homologs of *bHLH* and *TIP41* (Figure 1a). The variations in gene copy number and gene orientation indicated *CYP81Dx* cluster was dynamic and tended to occur interspecific duplication in grass genomes. A phylogenetic analysis implied that *TaCYP81D3* and *TaCYP81D4* represent the outcome of a duplication event specific to wheat (Figure 1b). The greater physical length of the *CYP81Dx* cluster in wheat likely reflects the high level of repetitive DNA characteristic of the wheat genome (IWGSC, 2014).

### The profile of *TaCYP81D5* transcription

**Figure 2:**
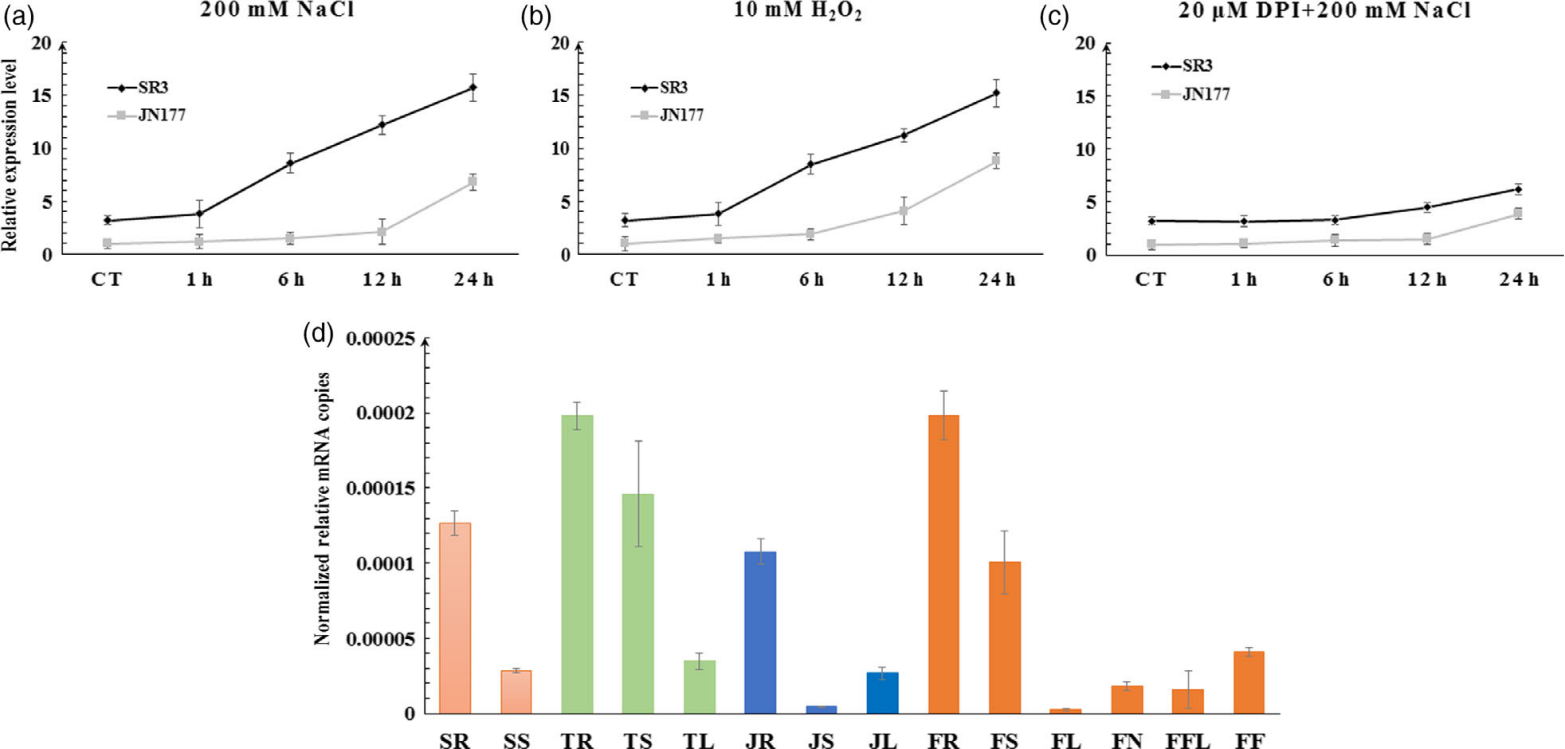
The profile of *TaCYP81D5* transcription. Transcript abundances are shown for both JN177 (grey line) and SR3 (black line) plants subjected to (a) salinity stress, (b) H_2_O_2_ stress, (c) salinity plus DPI. (d) The gene's transcription in various organs sampled at Zadoks stages Z11 (in orange), Z21 (green), Z32 (blue) and Z59 (dark orange). SR and SS: respectively, root and shoot samples taken at Z11; TR, TS and TL: respectively, root, shoot and leaf samples taken at Z21; JR, JS and JL: respectively, root, shoot and leaf samples taken at Z32; FR, FS, FL, FN, FFL and FF: respectively, root, stem, leaf, node, flag leaf and spike samples taken at Z59. *TaEF1‐*α (M90077) (Paolacci *et al*., 2009) was chosen as the endogenous control. Each bar represents the mean ± SD of at least three biological replicates.

A qRT‐PCR assay showed that four of the five *TaCYP81Dx* genes (the exception was *TaCYP81D1*, for which only a very low level of transcript was detected) were induced by salinity stress in the roots of both SR3 and JN177 (Figure S1b–e). Of these four genes, however, only *TaCYP81D5*, similar to the positive control *TaFLS1* (Figure S1f; Wang *et al*., 2014), showed a higher abundance of transcript in SR3 than in JN177, both in stressed and non‐stressed seedlings (Figure 2a). The gene was strongly transcribed in vegetative organs, and particularly so in the root of SR3 (Figure 2d). Meanwhile, compared to other four *TaCYP81Dx* genes, *TaCYP81D5* also showed the highest level of expression in roots of model bread wheat cultivar Chinese Spring (Figure S1g; Choulet *et al*., 2014). In JN177 roots, the abundance of the transcript was not greatly affected over the first 12 h of exposure of the roots to 200 mm NaCl, but by 24 h, the abundance of transcript rose to some sixfold the level present in non‐stressed roots; in contrast, in SR3 roots, *TaCYP81D5* was induced more rapidly (within 6 h; Figure 2a). When the transcriptional response of *TaCYP81D5* to the exposure to 10 mm H_2_O_2_ was investigated, a result similar to that induced by salinity stress was observed, in that the abundance of the transcript was higher in SR3 than in JN177, and the response was more rapid (Figure 2b).

One major biochemical basis for superior salt tolerance of SR3 against JN177 is the stronger ROS accumulation (Liu *et al*., 2014), which is consistent with the higher expression of *TaCYP81D5* in SR3. An additional provision of 20 μm DPI, an inhibitor of NADPH oxidase resulting in diminishing ROS productions, could largely counteract the induction of *TaCYP81D5* by salinity (Figure 2c), indicating that the expression of *TaCYP81D5* was mediated by ROS.

### Epigenetic events contribute to the transcription of *TaCYP81D5*

**Figure 3:**
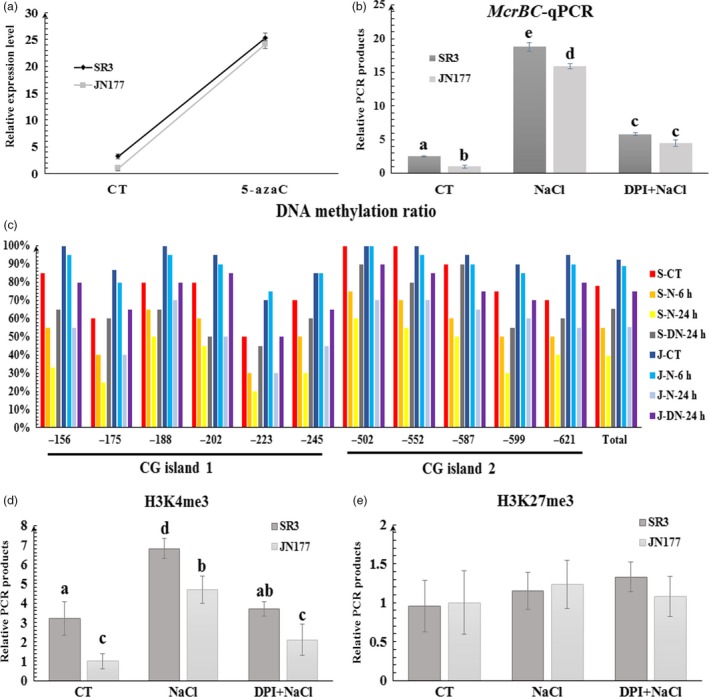
The epigenetic modification status of *TaCYP81D5* and its relationship to transcript abundance. (a) The transcription of *TaCYP81D5* in wheat roots treated with 5‐azaC. *TaEF1‐*α (M90077) (Paolacci *et al*., 2009) was chosen as the endogenous control. (b) *McrBC‐* qPCR analysis of *TaCYP81D5* in wheat roots. *TaWRKY40*, a gene without methylated modification, was used as a reference gene for *Mcr* BC‐qPCR (Wang *et al*., 2014). (c) Cytosine methylation ratio of *TaCYP81D5* in wheat roots. The numbers shown on the *x‐*axis refer to the base number counting from the start codon. (d) The H3K4me3 level of *TaCYP81D5* in wheat roots. (e) The H3K27me3 level of *TaCYP81D5* in wheat roots. S: SR3; J: JN177; CT: non‐stressed conditions; N: salinity‐stressed conditions; DN: salinity plus DPI treatment. Each bar represents the mean ± SD of at least three biological replicates. Different letters on the top of the bars indicate significance using the one‐way Waller–Duncan test (*P *<* *0.05).

In order to further investigate the cause of different expression level of *TaCYP81D5* between SR3 and JN177, the ~2000 bp upstream region and the gene‐body region of *TaCYP81D5* were amplified from SR3 and JN177, respectively, and sequenced. However, no genetic variation within the promoter and gene‐body regions of *TaCYP81D5* was discovered between these two cultivars. The expression of duplicated gene is prone to being regulated by epigenetic modifications (Deng *et al*., 2017). Moreover, our previous study has indicated multiple salinity‐responsive genes in wheat are epigenetic regulated (Wang *et al*., 2014). These clues encourage us to check whether the expression of *TaCYP81D5* is associated with epigenetic modifications. In seedlings exposed to 50 μm 5‐azaC, a DNA methyltransferase inhibitor resulting in DNA demethylation, the transcription of *TaCYP81D5* was significantly induced (Figure 3a). A comparison of the DNA methylation status of the *TaCYP81D5* promoter region in JN177 and SR3, achieved using bisulphite sequencing, showed that a higher level of methylation pertained in the JN177 than in the SR3 sequence (Figure 3c). A 6‐h exposure of SR3 seedlings to 200 mm NaCl was sufficient to reduce the methylation status of the *TaCYP81D5* sequence, but the process took 24 h in salinity‐challenged JN177 seedlings. The *Mcr*BC‐qPCR assay supported these observations (Figure 3b). Additionally, a restoration experiment showed that the expression level of *TaCYP81D5* was dramatically decreased when the salinity was removed for 48 h (Figure S2a), and the DNA methylation ratio was recovered (Figure S2c), which further confirmed the involvement of DNA methylation in the salinity response of *TaCYP81D5*.

DNA methylation is usually accompanied with other epigenetic modifications, including histone methylation and acetylation (Gutzat and Scheid, 2012). Inspection of the ChIP‐seq data (Qi *et al*., 2018; Ramírez‐González *et al*., 2018) suggested *TaCYP81D5* was highly modified by H3K4me3 and H3K27me3 (Figure S3a). Using ChIP‐qPCR assay, it was intriguingly discovered only the level of H3K4me3 in the 5′ upstream region, particularly around the transcriptional start site (Figures 3d and S3a–c), of *TaCYP81D5* was associated with the expression of *TaCYP81D5* under salt stress. The level of H3K27me3 (Figures 3e and S3d,e) of *TaCYP81D5* was similar to the situation of control gene, *TaSRO1* (Figure S3f–j; Liu *et al*., 2014; Wang *et al*., 2014), which was not affected by salinity.

The level of ROS content could affect the status of epigenetic modification (Franco *et al*., 2008). When exposed to 10 mm H_2_O_2_, the DNA methylation ratio of *TaCYP81D5* was decreased (Figure S2b,d). In the presence of 20 μm DPI, the effect of salinity on DNA methylation (Figure 3b,c) and H3K4me3 (Figure 3d) of *TaCYP81D5* was suppressed. These results indicated that the contribution of the epigenetic modification to the salinity induction of *TaCYP81D5* was associated with ROS accumulation.

### *TaCYP81D5* is deposited in the endoplasmic reticulum (ER)

The transient expression of *35S::TaCYP81D5*‐*GFP* in both onion epidermal cells and wheat protoplasts was used to reveal the site of TaCYP81D5 deposition (Figure S4). Whereas the signal derived from the control *35S::GFP* transgene appeared throughout the nucleus and cytoplasm, the TaCYP81D5‐GFP fusion protein was absent from the nucleus, instead being distributed in discrete regions of the cytoplasm (Figure S4a). Plant P450 proteins are usually anchored to ER or Golgi apparatus through a short hydrophobic segment of their N‐terminus (Bak *et al*., 2011). When the *35S::TaCYP81D5*‐*GFP* and BiP:RFP (a subcellular marker of ER) transgenes were co‐transferred, the GFP and RFP signals overlapped (Figure S4b,c), implying that TaCYP81D5 was deposited in the ER.

### *TaCYP81D5* contributes to wheat salinity tolerance

**Figure 4:**
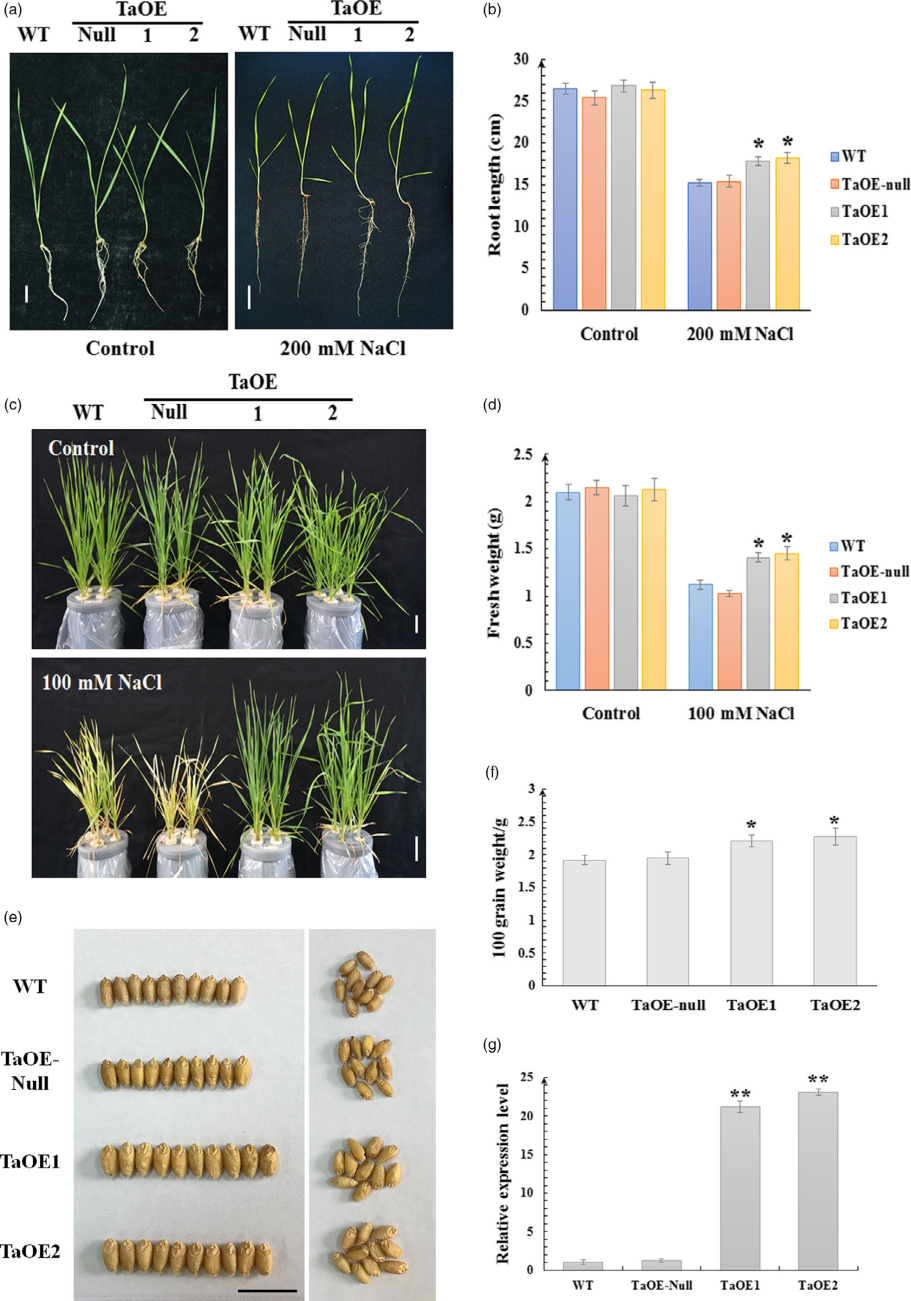
*TaCYP81D5* contributes to the salinity tolerance of wheat. (a) The seedling phenotype and (b) the root length of wild‐type (WT) wheat, TaOE‐null (null transgenic wheat lines) and TaOE1 and TaOE2 (transgenic wheat lines overexpressing *TaCYP81D5*) under control condition or a short‐term and high concentration (200 mm for 4 days) salt stress. (c)The seedling phenotype and (d) fresh weight of WT wheat, TaOE‐null, TaOE1 and TaOE2 under control condition or a long‐term and moderate concentration (100 mm for 15 days) salt stress. (e) The grain weight and (f) the grain size of WT wheat, TaOE‐null, TaOE1 and TaOE2 raised in moderate salinity soil‐filled pots. (g) The relative expression of *TaCYP81D5* in wild‐type, TaOE‐null, TaOE1 and TaOE2 wheat lines. *TaEF1‐*α (M90077) (Paolacci *et al*., 2009) was chosen as the endogenous control. Transcript abundance of *TaCYP81D5* in WT was calculated by giving the value 1. Data are presented as mean ± SE of at least three biological replicates. Columns marked with one asterisk indicate significant differences (*P *<* *0.05) using Student's *t*‐test, and double asterisks indicate significant differences (*P *<* *0.01). Bar: 1 cm.

*TaCYP81D5* was constitutively over‐expressed in bread wheat to explore its role in salinity stress (Figure 4). Among the transgenic lines engineered to constitutively express *TaCYP81D5* in bread wheat, the two most effective expressors (TaOE1 and TaOE2) were retained, along with a sib line which lacked the transgenic effect (TaOE‐null; Figure 4g). In the absence of salinity stress, there was no phenotypic difference between TaOE1, TaOE2, TaOE‐null and WT seedlings, but in the presence of 200 mm NaCl, TaOE1 and TaOE2 plants developed longer shoots and roots than did either TaOE‐null or WT seedlings (Figure 4a,b). When challenged with a long‐term moderate salt stress (100 mm NaCl for 15 days) since three‐leaf stage, TaOE‐null and WT seedlings became very wilted, while the TaOE1 and TaOE2 seedlings remained robust (Figure 4c,d). Furthermore, when grown in moderate salinity soil‐filled pots, TaOE1 and TaOE2 could produce larger seeds and higher yield than control lines (Figure 4e,f). *TaCYP81D5* was also heterologously expressed in Arabidopsis (Figure S5). When germinated on a medium containing NaCl, the transgenic seeds germinated more rapidly than the control seeds (Figure S5a,b), and the proportion of seedlings forming a green cotyledon was larger (Figure S5a). In 5‐day‐old seedlings exposed to salinity, the length of the roots formed by the transgenic plants was greater than that achieved by control seedlings (Figure S5c,d). These results concluded that *TaCYP81D5* could enhance the salinity tolerance in both wheat and Arabidopsis.

**Figure 5:**
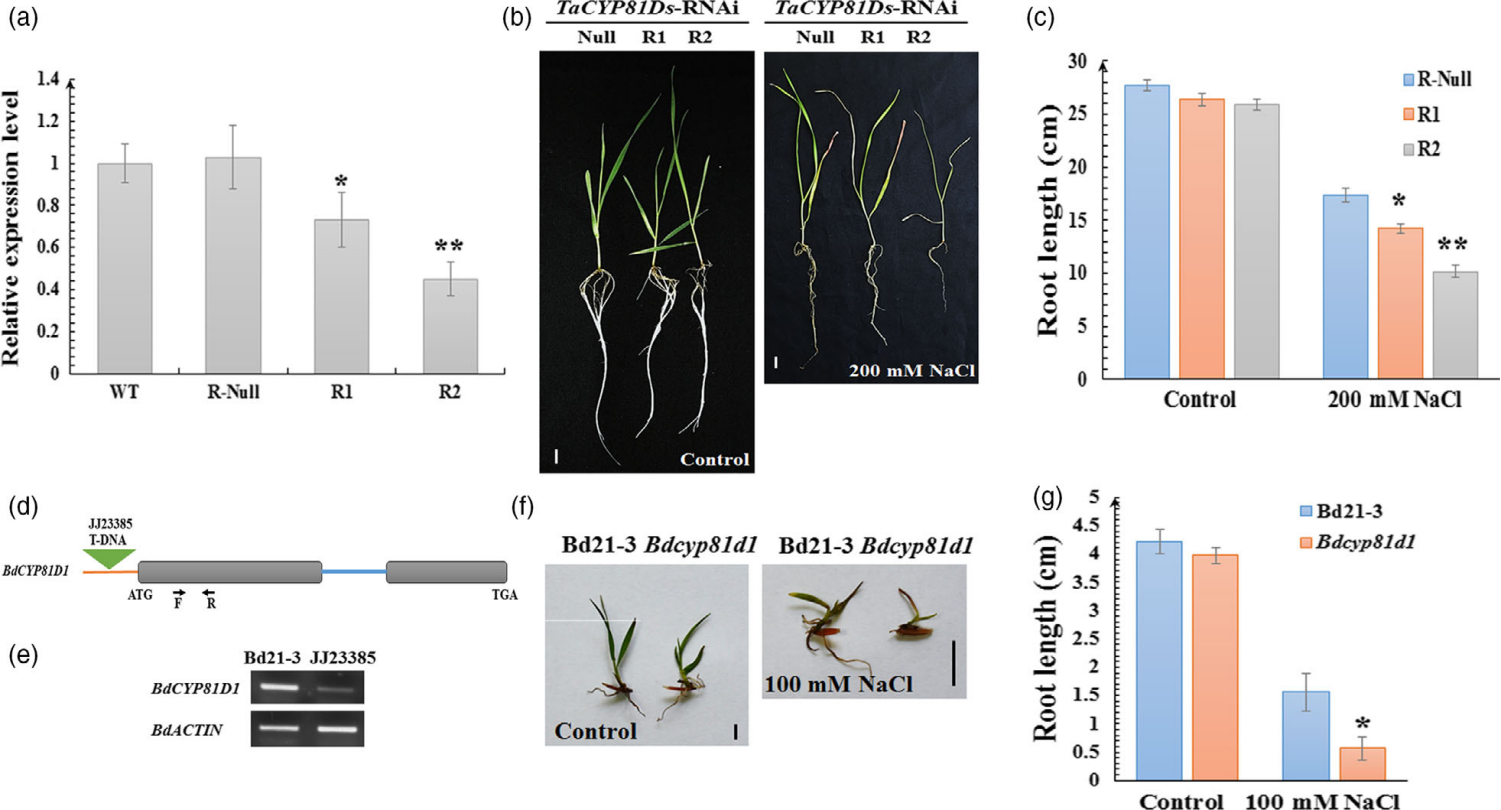
The effect on salinity tolerance of knocking down *TaCYP81Ds* and *BdCYP81D1*. (a) The relative expression of *TaCYP81Ds* in wild‐type, RNAi‐null, RNAi1 and RNAi2 wheat lines. *TaEF1‐*α (M90077) (Paolacci *et al*., 2009) was chosen as the endogenous control. Transcript abundance of *TaCYP81Ds* in WT was calculated by giving the value 1. (b) The seedling phenotype and (c) the root growth of *TaCYP81Ds* RNAi‐null line and RNAi lines. (d, e) The T‐DNA insertion in the 5′ UTR caused a knockdown of *BdCYP81D1* in *B. distachyon*. (f) The seedling phenotype and (g) the root growth of WT *B. distachyon* and the *Bdcyp81d1* mutant. Data are presented as mean ± SE of at least three biological replicates. Columns marked with one asterisk indicate significant differences (*P *<* *0.05) using Student's *t*‐test, and double asterisks indicate *P *<* *0.01. Bar: 1 cm. N.A.: no data available.

To further investigate the role of *TaCYP81D5* in salinity tolerance, the public loss‐of‐function mutants of *CYP81D5* (Figure S6a,b) in the background of tetraploid wheat cultivar Kronos were obtained. However, the seedling growth of *cyp81d5‐aabb* mutant was indistinguishable from that of Kronos itself under both normal and salinity‐stressed conditions (Figure S6c). Intriguingly, a higher abundance of both *CYP81D2* and *CYP81D4* transcript was discovered in the mutant (Figure S6d). Meanwhile, SALK_129086C, where T‐DNA was inserted into the promoter region of *AtCYP81D8*, one member of the *AtCYP81D* gene cluster in Arabidopsis (Figure 1a), was gained (Figure S6e,f). Once again, there was no clear phenotype associated with the mutation, at least in seedlings exposed to salinity stress (Figure S6g,h). These results imply that the other members in the *CYP81D* cluster may offer a buffering effect when one member is functionally deficient. To verify this hypothesis, RNAi lines of *TaCYP81D* genes in the cluster (*TaCYP81Ds*), based on the high‐sequence similarity among these *TaCYP81Dx* genes, were generated in the background of cv. SR3 (Figure 5a). Intriguingly, these RNAi knockdown lines showed more severe growth arrest than the control line under salinity stress (Figure 5b,c), and this effect was correlated with the expression level of *TaCYP81Ds* genes (Figure 5a). Meanwhile, a T‐DNA insertion mutant (*Bdcyp81d1*) of *BdCYP81D1*, which is the only *CYP81Dx* gene in the collinear region of *B. distachyon* (Figure 1a) and is also salinity‐inducible (Figure S1i), was obtained and analysed (Figure 5d,e; Thole *et al*., 2011). Under non‐stressed conditions, the mutant seedlings displayed slightly weaker growth, and when exposed to salinity stress, their growth was drastically compromised (Figure 5f,g). Moreover, as the expression levels of solely *TaCYP81D5* and total *TaCYP81Ds* were both higher in salt‐tolerant cv. SR3 than its salt‐sensitive parent cv. JN177 (Figures 2 and S1b–e), the F_2_ seeds of a cross between SR3 and JN177 were used for association study. The outcomes showed that both the expression levels of solely *TaCYP81D5* and total *TaCYP81Ds* were positive correlated with salinity tolerance (Figure S7a,b). Additionally, the expression levels of *TaCYP81Ds* were measured in selected bread wheat accessions with different salinity‐tolerant ability. Generally, the expression level of *TaCYP81Ds* in the 20 salinity‐tolerant accessions was the highest, while that in the 20 salinity‐sensitive accessions was the lowest (Figure S7c). These lines of evidence strongly suggest these duplicated *CYP81Dx* genes, as a cluster, contribute to wheat salinity tolerance cooperatively and redundantly, and solely knocking out/down one member cannot affect the salinity tolerance.

### *TaCYP81D5* accelerated the scavenging of ROS stimulated by salinity stress

**Figure 6:**
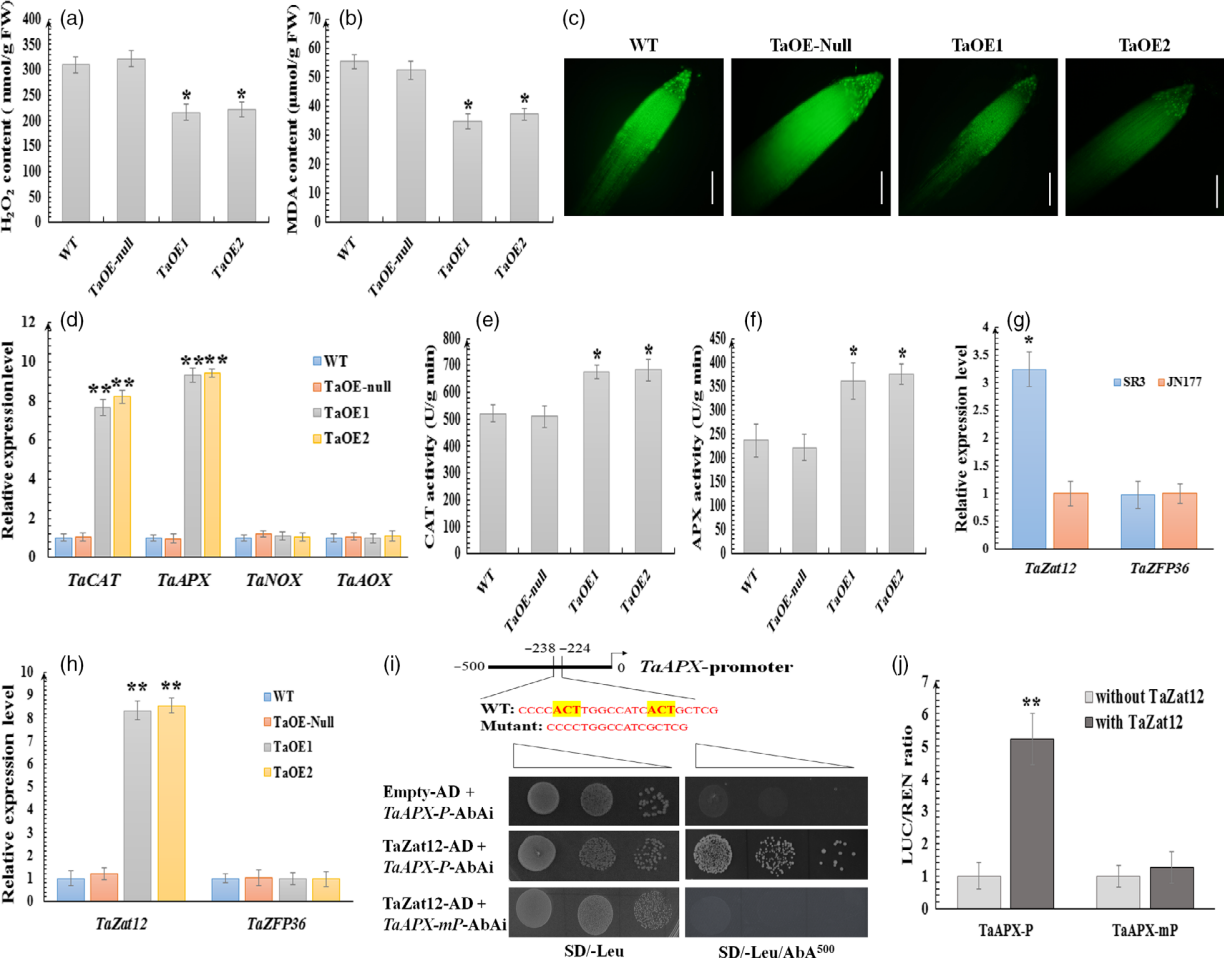
*TaCYP81D5* confers salinity tolerance by promoting Zat12‐mediated ROS signalling pathway, thereby enhancing ROS scavenging. (a) The H_2_O_2_ and (b) MDA contents in WT, TaOE‐null, TaOE1 and TaOE2 wheat lines. (c) ROS levels of WT, TaOE‐null, TaOE1 and TaOE2 wheat lines by carboxy‐H_2_ DCFDA staining. Bar: 0.2 cm. (d) The abundance of *TaCAT*,* TaAPX*,* TaNOX* and *TaAOX* transcript in roots of WT, TaOE‐null, TaOE1 and TaOE2 wheat lines. *TaEF1‐*α (M90077) (Paolacci *et al*., 2009) was chosen as the endogenous control. Relative transcript abundance of every gene in WT was calculated by giving the value 1. (e) CAT and (f) APX activities of WT, TaOE‐null, TaOE1 and TaOE2 wheat lines. (g) The transcript abundance of *TaZat12* and *TaZFP36* in roots of SR3 and JN177. Relative transcript abundance of every gene in JN177 was calculated by giving the value 1. (h) The transcript abundance of *TaZat12* and *TaZFP36* in roots of WT, TaOE‐null, TaOE1 and TaOE2 wheat lines. Relative transcript abundance of every gene in WT was calculated by giving the value 1. (i) Y1H assay showing that TaZat12 could bind the A(G/C)T repeats element in *TaAPX* promoter. *TaAPX‐P*: the ~500‐bp promoter fragment of *TaAPX*;* TaAPX‐mP*: a fragment deleting the A(G/C)T repeats element; SD/‐Leu, SD medium without Leu; SD/‐Leu/AbA^400^, SD medium without Leu supplemented with AbA at the concentration of 400 ng/mL. Transformed yeast cells were dotted at 10^−1^ dilutions on the selective medium. (j) Transient expression assay showing that TaZat12 could activate the expression of *TaAPX*, and the A(G/C)T repeats element is essential for this activation. The black column represents the relative LUC/REN ratio of the reporter in the present of empty effector (pBI221 empty vector) without TaZat12; and the grey column represents the relative LUC/REN ratio of the reporter in the present of effector containing TaZat12. Data are presented as mean ± SE of at least three biological replicates. Columns marked with one asterisk indicate significant differences (*P *<* *0.05) using Student's *t*‐test, and double asterisks indicate *P *<* *0.01.

Considering the stronger expression in SR3, an elevated ROS content cultivar and the regulation of ROS in salt‐responsive expression of *TaCYP81D5*, it prompted us to investigate whether ROS homeostasis was involved in *TaCYP81D5*‐mediated salinity tolerance. Wheat over‐expressors of *TaCYP81D5* exposed to 150 mm NaCl for 24 h accumulated at least 20% less H_2_O_2_ and malondialdehyde (MDA, an indicator of intracellular ROS damage) than was managed by either the TaOE‐null line or WT (Figure 6a,b). The tissue ROS content in the transgenics, as visualized by carboxy‐H_2_DCFDA staining, was demonstrably lower than in either TaOE‐null or WT (Figure 6c). To determine the molecular mechanism of lower ROS level in *TaCYP81D5* overexpression lines, the expression of wheat genes related to ROS production and scavenging was examined. As shown in Figure 6d, the abundance of both *TaCAT* and *TaAPX* was higher in the over‐expressors, while that of the ROS synthesis genes *TaNOX* and *TaAOX* (Table S1) was comparable. The measured activity of APX and CAT in TaOE1 and TaOE2 was, respectively, 50% and 20% higher than that present in either TaOE‐null or WT (Figure 6e,f). Meanwhile, in *A*. *thaliana* plants expressing *TaCYP81D5*, a lower ROS content and a higher transcription of *AtCAT1*,* AtCAT2* and *AtAPX1*, as well as the enzyme activity of CAT and APX, were also discovered (Figure S8), indicating that *TaCYP81D5* enhanced salinity tolerance in bread wheat mainly through accelerating ROS scavenging.

### *TaCYP81D5*'s influence over ROS scavenging‐dependent stress signalling requires Zat12

To examine the mechanism how *TaCYP81D5* influences ROS scavenging, the expression levels of a set of well‐known genes involved in stress‐responsive signalling pathways were compared between WT and AtOX lines. The outcome showed that the abundance of *AtZat12* transcript (Table S1), encoding a C2H2 zinc finger transcription factor required for the induction of *AtAPX1* and the transduction of ROS signal (Rizhsky *et al*., 2004), was significantly higher in the AtOX lines (Figure S9a). According to Davletova *et al*. (2005), the *A*. *thaliana zat12* mutant is salinity‐sensitive, and the level of *AtAPX1* transcription is rather lower in *zat12* than in WT plants, irrespective of whether or not the plants are exposed to salinity stress. When *TaCYP81D5* was expressed in *zat12* mutant, the root length was superior to that of *zat12* seedlings, but inferior to that of WT seedlings, under 120 mm NaCl treatment (Figure S9b,c). The abundance of *AtAPX1* transcript in the *TaCYP81D5* expressors was 4.5‐fold higher than that in the *zat12* mutant, but rather lower than that in WT (Figure S9d).

*Zat12* has not been isolated in bread wheat up to our knowledge, while the homologous gene in rice, *OsZat12*, was identified (Imran *et al*., 2016). Using *OsZat12* as a query sequence, *TaZat12* was found in the newly released wheat genome (Table S1). Moreover, *TaZFP36*, a homologous gene of another rice zinc finger transcription factor *OsZFP36*, which was reported as a transcriptional activator of *OsAPX1* (Huang *et al*., 2018), was also isolated (Table S1). The transcript abundance of *TaZat12* was greater in SR3 than in JN177, while that of *TaZFP36* was similar between these two cultivars (Figure 6g). Furthermore, *TaZat12* also showed a higher expression level in wheat over‐expressors of *TaCYP81D5*, while *TaZFP36* did not (Figure 6h). Using yeast one‐hybrid (Y1H) assay, TaZat12 was proved to be able to bind the promoter of *TaAPX* (Figure 6i). Transient expression assay was then used to check the influence of TaZat12 on *TaAPX* expression. As shown in Figure 6j, the LUC signal was stronger in the presence of TaZat12, suggesting TaZat12 could directly activate the expression of *TaAPX*. Zat12 belongs to a family of abiotic stress‐responsive C2H2‐type zinc finger proteins, among which the binding sites of AZF1, AZF2 and AZF3 in Arabidopsis are all confirmed as the A(G/C)T repeats element (Sakamoto *et al*., 2004). An A(G/C)T repeats element in the −242 to −229 bp region from the start codon of *TaAPX* was discovered (Figure 6i). When these two A(G/C)T repeats elements were deleted, the abilities of TaZat12 to bind the promoter of *TaAPX* and activate its expression were abolished (Figure 6i,j). The interpretation of these results was that the ability of TaCYP81D5 to promote ROS scavenging is, at least in part, dependent on Zat12.

## Discussion

### The evolution of plant *CYP81* genes

CYP81 family is an A type P450 subfamily which is specific to plants (Bak *et al*., 2011). Intriguingly, the size of this subfamily is prone to expanding due to gene tandem duplication, which can generate new *CYP* gene showing distinct or cooperative function (Nelson and Werck‐Reichhart, 2011). In *A. thaliana*, the products of the tandemly arranged *CYP81F1*,* CYP81F3* and *CYP81F4* are all able to convert indol‐3‐yl‐methyl to 1‐methoxy‐indol‐3‐yl‐methyl, whereas only CYP81F1 and CYP81F3 can catalyse the conversion of indol‐3‐yl‐methyl to 4‐hydroxy‐indol‐3‐yl‐methyl (Bednarek *et al*., 2009; Pfalz *et al*., 2011). Here, an array of five wheat *CYP81D* genes was located within a genomic region associated with the salinity response (IWGSC, 2014; Wang *et al*., 2018); in the corresponding region of the *B. distachyon* genome (IBI, 2010), there exists only a single *CYP81D* gene, while four copies are present in the genomes of rice (Goff *et al*., 2002), sorghum (Paterson *et al*., 2009) and barley (IBGSC, 2012; Figure 1a). In Arabidopsis, five *AtCYP81Dx* genes (*AtCYP81D2‐5* and *AtCYP81D8*) are in tandem configuration on chromosome 4 (Bak *et al*., 2011); however, the gene orientation and flanking genes were different from wheat (Figure 1a). These variations in copy number, gene orientation and flanking genes conclude *CYP81Dx* genes are dynamic and tend to be duplicated during evolution. Moreover, *TaCYP81D3* and *TaCYP81D4* showed the highest similarity and a neighbouring distribution (Figure 1a,b), suggesting these genes are generated by an intraspecific duplication in wheat.

Both the *Medicago truncatula MtCYP81Es* (Liu *et al*., 2003) and *Cucumis sativus CsCYP81Qs* (Shang *et al*., 2014) are up‐regulated by salinity stress. Of the set of *A*. *thaliana CYP* genes, *CYP81D8* seems to be the most responsive to salinity stress (Narusaka *et al*., 2004). These evidences suggest members of *CYP81* subfamily are conserved in the aspect of salinity response among plant species. In wheat, four of the five *CYP81D* genes are strongly induced by salinity stress (Figure S1); their tandem arrangement makes it likely that the members of the cluster act redundantly in terms of the plant's salinity response. Experimental confirmation of their redundancy was obtained by showing that the mutation of *TaCYP81D5* had no detrimental effect on the level of the plant's salinity tolerance (Figure S6a–c), while knocking down of the expression of the total *TaCYP81D* cluster caused a salinity‐sensitive phenotype (Figure 5a–c). Moreover, knocking down the expression of the non‐duplicated *B. distachyon* gene *BdCYP81D1* (Figure 1a) also increased the plant's sensitivity to the stress (Figure 5f,g). *B. distachyon* is a closely related species to Triticeae (Wicker *et al*., 2011). Given the importance of the *CYP81Dx* genes to the salinity tolerance of wheat (Figures 4a–f and 5b,c), the suggestion is that the evolved variation in copy number and their functional redundancy have provided wheat with the means to combat soil salinity more successfully than is possible for its relative *B. distachyon*.

### The nexus between salinity stress, ROS accumulation and DNA methylation in regulating the expression of *TaCYP81D5*

**Figure 7:**
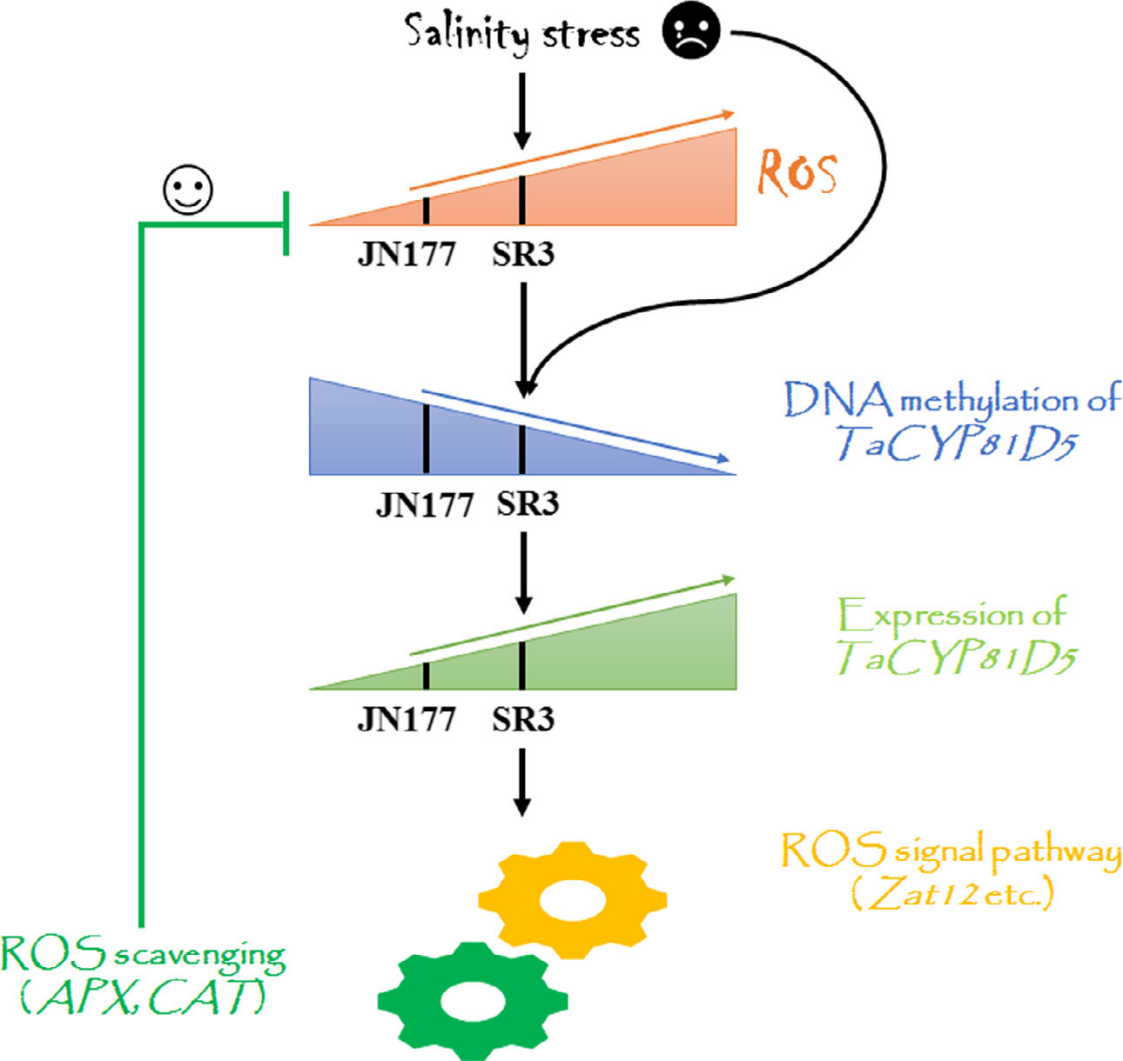
A proposed model of the contribution made by TaCYP81D5 to the salinity tolerance of wheat. In plants encountering salinity stress, ROS tend to accumulate, which may impact the chromatin modification status of *TaCYP81D5* in a way which favours its transcription. In cultivars such as SR3, which are able to accumulate a particularly high content of ROS,* TaCYP81D5* is more strongly expressed than it is in weaker accumulators, such as the cultivar JN177. The induction of *TaCYP81D5* in turn up‐regulates genes encoding both ROS signal transduction proteins and ROS scavenging enzymes in order to restore ROS homeostasis, thereby acting to enhance the plants’ salinity tolerance.

ROS accumulation is a common plant response to salinity stress (Munns and Gilliham, 2015). Under both stressed and non‐stressed growing conditions, the tissue content of ROS in SR3 is higher than in JN177 (Liu *et al*., 2014), with consequences for the level of expression of certain salinity‐responsive genes (Liu *et al*., 2012), forming the major biochemical and genetic basis for superior salt tolerance of SR3 (Wang and Xia, 2018). The abundance of *TaCYP81D5* transcript in SR3 was positively correlated with its tissue ROS content (Figure 2a), and the gene was more rapidly induced by the stress than in JN177 (Figure 2a), suggesting in SR3 with a higher background level of ROS, the ROS concentration reached to the threshold more rapidly to trigger *TaCYP81D5*. When plants were treated with the ROS accumulation inhibitor compound DPI, there was a marked suppression of *TaCYP81D5*'s induction by salinity (Figure 2c). Given that the product of *AtCYP81D8* has been recognized as a marker gene of the oxidative stress response (Baruah *et al*., 2009), the conclusion is that the salinity‐induced up‐regulation of *CYP81D* genes is typically mediated by ROS accumulation (Figure 7).

Another marked difference between the genomes of SR3 and JN177 is the extent of the epigenetic differences present, most notably those involving DNA methylation (Wang *et al*., 2014), which are thought to be a side‐effect of the somatic hybridization procedure used to derive SR3 (Liu *et al*., 2015). DNA methylation variants are known to generate transcriptional changes in a range of salinity stress‐responsive genes, including *TaFLS1* (Wang *et al*., 2014) and *TaTIP2;2* (Xu *et al*., 2013). The behaviour of *TaCYP81D5*, namely its higher level and more rapidly induced transcription in SR3 (Figure 2a,b), is associated with DNA methylation status (Figure 3b,c), providing a further example of this phenomenon. Moreover, DNA methylation is usually accompanied with other chromatin modifications, including histone methylation and acetylation (Gutzat and Scheid, 2012). The histone modification, at least H3K4me3, is found also involved in the regulation of *TaCYP81D5* (Figure 3d). There is evidence that, in mammalian genomes, the accumulation of ROS can of itself induce novel epialleles (Franco *et al*., 2008). Treatment with DPI, which suppresses ROS synthesis, had the effect of inhibiting the DNA demethylation and H3K4me3 of *TaCYP81D5* induced by salinity stress, thereby counteracting the up‐regulation of *TaCYP81D5* (Figure 3b–e). These results conclude a regulatory mechanism of *TaCYP81D5* under salt stress that the elevated ROS content in SR3 or under salinity treatment will cause the epigenetic rearrangement and then induce *TaCYP81D5* (Figure 7). However, the mechanism underlying this phenomenon, for example, whether ROS can affect the expression and/or enzyme activity of DNA methyltransferase and/or demethylase, needs further investigations.

### The role of *TaCYP81D5* in the determination of salinity tolerance in wheat

ROS not only activate the salinity stress response pathway, but also damage DNA, enzymes and lipids; thus, their accumulation has to be strictly regulated (Baxter *et al*., 2013; Yang and Guo, 2018). ROS homeostasis is maintained through a balance between ROS production and ROS scavenging (Mittler *et al*., 2004). In *A*. *thaliana*, the upstream regulatory factors encoded by *Zat7*,* Zat10* and *Zat12* represent a key component of this process (Mittler *et al*., 2011). In SR3, *TaSRO1*, a gene encoding a poly (ADP ribose) polymerase (PARP) domain protein, is essential for ROS homeostasis through a refined regulation involving enhancements of ROS production via NOX and AOX enzyme system and also ROS scavenging via enzymes of the GPX cycle (Liu *et al*., 2014). The product of *TaOPR1*, a further gene carried by SR3, has been shown to support the expression of both *CAT* and *APX*, hence influencing the level of enzyme‐based antioxidant activity in the plant (Dong *et al*., 2013). When *TaCYP81D5* was constitutively expressed in either bread wheat or *A*. *thaliana*, the effect was to raise the plants’ level of salinity tolerance, which was not only during germination and early seedling growth but also at the reproductive stage (Figure 4). The cellular content of both ROS and MDA was lower in these transgenic plants than in their WT equivalents (Figures 6a–c and S8c–e). The reduction in ROS accumulation was largely due to the up‐regulation of *CAT* and *APX* genes (Figures 6d and S8f), leading to a higher activity level of their encoded enzymes (Figures 6e,f and S8g,h), whereas genes related to ROS production were not affected (Figure 6d). The key ROS signal transduction gene *Zat12* was up‐regulated in *A. thaliana* plants constitutively expressing *TaCYP81D5* (Figure S9a), while in the absence of a functional copy of *AtZat12*, the level of induction of both *CAT* and *APX* was significantly attenuated (Figure S9d). In *TaCYP81D5* overexpression wheat lines, the expression of *TaZat12* could be enhanced (Figure S9d). Meanwhile, *TaZat12* could bind the A(G/C)T repeats element in promoter of *TaAPX* and activate its expression (Figure 6i,j), coinciding with the stronger expression of *TaAPX* in constitutive wheat expressors of *TaCYP81D5*. These results support that *TaCYP81D5* confers salinity tolerance in bread wheat largely through an enhancement of ROS signal transduction and scavenging (Figure 7).

Given the important role of *TaCYP81D5* in salinity tolerance, it will be interesting to investigate how TaCYP81D5, an ER deposited protein (Figure S4), affects the expression levels of genes related to the ROS signal transduction and scavenging. Generally, as versatile catalysts, almost all of the plant P450 proteins are anchored to ER or Golgi apparatus to play essential roles in the biosynthesis of different primary and secondary metabolites. In many cases, molecules produced by P450 proteins can act as a signal to trigger a transcriptomic rearrangement. More importantly, some molecules produced by P450 proteins are able to interfere with proteins or DNA, thus causing a direct signalling response, which was mainly clarified in animals (Nebert and Dalton, 2006). There are increasing lines of evidence suggesting that similar effects of P450 proteins may also exist in plants (Mizutani and Ohta, 2010). Therefore, future investigations, to identify the exact metabolite produced by TaCYP81D5 and to reveal the potential contribution of this metabolite to salinity tolerance of wheat, may not only bridge the gap between *TaCYP81D5* and *TaZat12*, but also offer a meaningful metabolic target for improving salinity tolerance of crops.

## Materials and Methods

### Identification of a cluster of salinity‐responsive *TaCYP81Dx* genes and their phylogenetic relationship

Inspection of our archival microarray data (Liu *et al*., 2012) showed that the probe ta_06616, marking a *CYP81D* sequence, was each significantly induced by salinity stress, and that their abundance in the transcriptome differed between SR3 and JN177. The probe sequence was used to screen cDNA libraries created in both SR3 and JN177, resulting in the isolation of the sequence *TaCYP81D5*. The *TaCYP81D5* locus lies on the long arm of chromosome 5B, within a cluster of five salinity‐responsive *TaCYP81Dx* genes (Zhang *et al*., 2016), namely TraesCS5B01G402700, TraesCS5B01G402800, TraesCS5B01G402900, TraesCS5B01G403000 and TraesCS5B01G403100 (Table S1). The IWGSC reference sequence v1.0 (http://www.wheatgenome.org/) was used to provide the genomic sequences of these genes, including both their coding and promoter sequences, which were used as a basis for amplifying and then resequencing the copies present in both SR3 and JN177. The relevant primer sequences are given in Table S2.

Five *CYP81Dx* genes in wheat mentioned above, and collinear *CYP81Dx* genes in other representative grass species including rice and sorghum, in wheat relative species including *Brachypodium distachyon* and barley, and in Arabidopsis, were chosen to generate the phylogenetic tree. The deduced polypeptide sequences of these CYP81Dx, following their alignment based on the ClustalW algorithm (http://www.clustal.org), were subjected to a phylogenetic analysis, applying the neighbour‐joining method (Saitou and Nei, 1987). The analysis used routines implemented in MEGA v6 software (http://www.megasoftware.net).

### Plant materials and growing conditions

The bread wheat materials including cultivars SR3, JN177 and the F_2_ seeds of a cross between SR3 and JN177 were stored in our laboratory. A panel of 307 bread wheat accessions for the natural variation identification and association study shared from Prof. Zhensheng Kang's group (Northwest A&F University, China). The wheat mutant, *cyp81d5‐aaBB* (Kronos2900 in Kronos background) and *cyp81d5‐AAbb* (Kronos3558; Figure S6a,b), was generated by Krasileva *et al*. (2017) and ordered from the Chinese distribution site, Shandong Agricultural University. By crossing, the double mutant, *cyp81d5‐aabb*, was obtained. To generate a construct containing a transgene able to over‐express *TaCYP81D5*, the coding sequence present in SR3 was amplified and inserted into a modified pGA3626 vector under the control of the maize ubiquitin promoter (Kim *et al*., 2009). To generate the RNAi lines of *TaCYP81Ds*, the sense and antisense fragments covering the conserved region of *TaCYP81D2‐5* (*TaCYP81D1* was silent according to Figure S1b,g) were inserted into a *ZmUbiquitin*‐promoter‐containing vector, pTCK303 (Liu *et al*., 2014). The constructs were transformed into the salinity‐sensitive cv. JN17 (for overexpression) or the salinity‐tolerant cv. SR3 (for RNAi) via shoot apical meristem method (Liu *et al*., 2014).

Wheat seedlings were raised hydroponically in half‐strength Hoagland's liquid medium (pH 6.0) which was replaced every 2 days until the plants had reached the three‐leaf stage. The medium was then adjusted to contain one of either 200 mm NaCl, 10 mm H_2_O_2_, 200 mm NaCl/20 μm of the NADPH oxidase inhibitor DPI (diphenyleneiodonium) or 50 μm of the DNA methyltransferase inhibitor 5‐azaC (5‐azacytidine; Wang *et al*., 2014), and the seedlings were allowed to grow for a further 1, 6, 12 h or 24 h. To avoid the effect of photoperiod, the seedlings were treated at different time points to make sure that the sampling time point was the same (Figure S1h). For restoration experiment, an additional 48‐h restoring treatment after salinity or H_2_O_2_ treatment was performed. The effect of salinity stress was measured after either a 4‐day exposure of three‐leaf stage seedlings to either 200 mm NaCl (before which the treatment was applied by the daily addition of 50 mm NaCl until the concentration had reached 200 mm) or a 15‐day exposure to 100 mm NaCl. The condition of the chamber was 14‐h/10‐h light/dark under the temperature 22/20 °C, a relative humidity of 50% and 300 μmol m^−2^ s^−1^ PAR (photosynthetically active radiation). To check the ultimate effect of salinity, seedlings germinated on moist filter paper at 20 °C were raised in moderate salinity soil‐filled pots and held in a growth chamber under a 12‐h photoperiod, a day/night temperature regime of 22/20 °C, a relative humidity of 50% and a light intensity of 300 μmol m^−2^ s^−1^. The moderate salinity soil was collected from Dongtai beach experimental station (Jiangsu Province, China), and the initial total soluble salts per 100 g dry soil were ~0.24 g. For spatial expression analysis, the plants grown in normal soil‐filled pots were sampled at the following Zadoks scale stages (Zadoks *et al*., 1974): seedling stage (Z11), tillering stage (Z21), jointing stage (Z32) and the flowering period (Z59).

In addition to the *A. thaliana* wild type (Col‐0 ecotype), the experiments used the two mutants *Atcyp81d8* (SALK_129086C) and *atzat12* (SALK_037357), obtained from the Arabidopsis Biological Resource Center (https://abrc.osu.edu/). Transgenic *TaCYP81D5* constitutive expression lines were generated by introducing the construct *pSTART::TaCYP81D5*, using the floral dip method. A germination assay was conducted in which surface‐sterilized seeds were plated on solidified half‐strength Murashige and Skoog medium (½ MS) containing either 0, 100 or 140 mm NaCl. The plates were held in the dark at 4 °C for 3 days and then exposed to a 16‐h photoperiod (light intensity, 200 mm m^−2^ s^−1^), a constant temperature of 22 °C and a constant relative humidity of 70%. To assay seedling phenotypes, 5‐day‐old seedlings raised on solidified ½ MS medium were re‐plated onto solidified ½ MS agar medium containing either NaCl (0, 80 or 120 mm) or H_2_O_2_ (0, 0.5 or 1.5 mm) under the same environmental conditions and were scored after 10 days. All experiments were performed in triplicate.

### RNA extraction and transcriptional profiling

Total RNA was extracted utilizing the TRIzol reagent (TaKaRa), and the first cDNA strand was synthesized using a PrimeScript™ RT Reagent Kit, along with gDNA Eraser (TaKaRa). The resulting cDNAs provided the template for a quantitative real‐time PCR (qRT‐PCR) assay, based on the SYBR^®^ Premix Ex Taq™ II reagent (TaKaRa). *TaEF1‐*α (M90077) (Paolacci *et al*., 2009) was chosen as the reference gene for the transcriptional profiling of wheat samples and *AtActin2* (*At3 g18780*) for those of *A*. *thaliana*. Estimates of transcript abundance were based on four technical replicates made from each of three biological replicates. The relevant primer sequences are given in Table S2.

### Bisulphite sequencing

Genomic DNA, isolated from the same samples used to extract RNA, was processed for bisulphite sequencing using an EpiTect Bisulfite Kit (Qiagen), following the manufacturer's protocol. The sequences of the necessary primers, designed using MethPrimer software (Li and Dahiya, 2002), are shown in Table S2. Both the experimental procedures and the analysis of the data followed the suggestions made by Wang *et al*. (2014).

### *Mcr*BC‐qPCR

*Mcr*BC is an endonuclease that specifically digests methylated but not unmethylated DNA. After *McrBC* treatment, methylated DNA will be cut and therefore cannot be amplified by PCR. Genomic DNA was extracted from seedlings utilizing cetyltrimethylammonium bromide (CTAB) method. A 1 mg aliquot of the resulting DNA was digested with 20 U *Mcr*BC restriction endonuclease (New England Biolabs) for 3 h in a 50 μL reaction. The subsequent amplification procedure was carried out using a Cycler 480 real‐time PCR system (Roche Diagnostics, Mannheim, Germany), following the manufacturer's instructions. The reference sequence was *TaWRKY40*, a gene which is generally not methylated (Wang *et al*., 2014). Estimates of the amplicons’ abundance were based on four technical replicates made from each of three biological replicates. The relevant primer sequences are given in Table S2.

### Chromatin immunoprecipitation (ChIP)‐qPCR assay

Previous ChIP‐seq data indicated *TaCYP81D5* was highly modified by H3K4me3 and H3K27me3 (Qi *et al*., 2018; Ramírez‐González *et al*., 2018) (which is visual on the Triticeae Multi‐omics Center: http://202.194.139.32/). ChIP with the antibodies H4K3me3 and H3K27me3 was performed as described (Zhang *et al*., 2012) with small modifications. For each assay, approximately 10 g fresh root samples were used. Chromatin precipitated without antibody and isolated chromatin before precipitation were used as negative control and input control, respectively. *TaEF1‐*α (M90077) was chosen as a control. *TaSRO1*, a salinity‐inducible gene (Liu *et al*., 2014) which was not regulated by DNA methylation (Wang *et al*., 2014), was also chosen as a control. Estimates of the amplicons’ abundance were based on four technical replicates made from each of three biological replicates. Based on the ChIP‐seq data, primers of three enriched regions of H3K4me3 and H3K27me3 were designed for *TaCYP81D5* (Table S2).

### Subcellular localization of *TaCYP81D5*

The *35S::TaCYP81D5*‐*GFP* construct was generated by inserting the *TaCYP81D5* coding sequence (without its stop codon) into the pBI221‐*GFP* plasmid. A subcellular marker of endoplasmic reticulum (ER), BiP:RFP, was shared from Inhwan Hwang group in Pohang University of Science and Technology, Korea. The *35S::TaCYP81D5*‐*GFP* and BiP:RFP constructs were co‐transferred into white onion epidermal cells or wheat protoplasts following Liu *et al*. (2014). After a 16‐h incubation at 22 °C in the dark, GFP/RFP‐generated fluorescence was detected using both bright‐field and fluorescence microscopy (FluoView 1000; Olympus; Japan).

### Quantification of tissue ROS content and antioxidant enzyme activity

Quantifications of H_2_O_2_ and malondialdehyde (MDA) content of sampled tissues were performed following the methods given by Liu *et al*. (2014). The ROS content of *A*. *thaliana* seedling samples was estimated following 3,3′‐diaminobenzidine (DAB) staining, following Dong *et al*. (2013), while for wheat root samples, carboxy‐H_2_DCFDA (2′,7′‐dichlorofluorescin diacetate) (Invitrogen, Carlsbad, CA) staining was used; these samples were incubated in 20 μm carboxy‐H_2_DCFDA at 37 °C for 30 min in the dark, rinsed in phosphate‐buffered saline and then subjected to fluorescence microscopy (Bx51; Olympus; Japan), applying an excitation wavelength of 488 nm and an emission wavelength of 522 nm. Quantification of the activity of ascorbate peroxidase (APX) and catalase (CAT) was achieved following methods given by Liu *et al*. (2014).

### Yeast one‐hybrid assay

The ~500‐bp promoter fragment of *TaAPX* or a fragment deleting the A(G/C)T repeats element (Figure 6i) was cloned into the pAbAi vector as a bait construct and then transformed into Y1HGold yeast strain (2nd lab™). The coding region of *TaZat12* was fused into the pGADT7 vector as a prey construct. The prey construct and the empty construct were separately transformed into the bait strain. The transformed yeast cells were grown at 30 °C for 4 days on the SD plates lacking Leu with or without antibiotic.

### Transient expression assay

To generate the reporter construct, the ~500‐bp promoter fragment of *TaAPX* or a fragment deleting the A(G/C)T repeats element was cloned into the pGreenII 0800‐Luc vector. To generate the *35S::TaZat12* effector construct, the coding region of *TaZat12* was fused into the pBI221 vector. The empty vector was used as a control. Transient expression assay was performed in *Arabidopsis* mesophyll cell protoplast, and the protoplast was isolated following methods given by Liu *et al*. (2014). The reporter construct with or without the effector construct was transformed into the protoplast via PEG‐mediated transformation. Then, the protoplast was incubated overnight. The relative LUC activity was determined by LUC/REN ratio using Dual‐Luciferase Reporter Assay Kit (Promega, Madison, WI) and a Synergy 2 multimode microplate (BioTek, Winooski, VT) according to the manufacturer's instructions.

## Conflict of interest

All the authors declare no conflict of interest.

## Author contributions

M.W. and S.L. planned and designed the research; M.W. performed most of the experiments and analysed the data in Jinan and Nanjing; J.Y. helped to perform wheat and Arabidopsis transformations; L.Q. helped to perform the subcellular localization and DNA methylation assay; and M.W., W.S., G.X. and S.L. wrote the article.

## Supporting information


**Figure S1** The transcriptional profiles of *CYP81Dx* genes.
**Figure S2** The transcriptional abundance and DNA methylation ration of *TaCYP81D5* in response to abiotic stress and the subsequently restoring treatment.
**Figure S3** Histone modifications of *TaCYP81D5* and the control gene *TaSRO1*.
**Figure S4** Subcellular localization of the TaCYP81D5‐GFP fusion protein.
**Figure S5** *TaCYP81D5* contributes to the salinity tolerance of *A*. *thaliana*.
**Figure S6** The effect on salinity tolerance of mutagenizing *CYP81Dx*.
**Figure S7** The contribution of *CYP81D* genes to salinity tolerance in additional genetic materials.
**Figure S8** The involvement of *TaCYP81D5* in H_2_O_2_ tolerance and ROS scavenging in Arabidopsis.
**Figure S9** Evidence of the involvement of Zat12 in the contribution made by TaCYP81D5 to salinity tolerance in Arabidopsis.

**Table S1** List of genes used in this study
**Table S2** Primers used in this study
