# Supplementary material for: TaCYP81D5, one member in a wheat cytochrome P450 gene cluster, confers salinity tolerance via reactive oxygen species scavenging
Source: Plant Biotechnol J. 2019 Sep 17;18(3):791–804. doi: 10.1111/pbi.13247 (PMC7004906; doi:10.1111/pbi.13247)
Supplement: Supplementary file 1 — Figure S1 The transcriptional profiles of CYP81Dx genes. Figure S2 The transcriptional abundance and DNA methylation ration of TaCYP81D5 in response to abiotic stress and the subsequently restoring treatment. Figure S3 Histone modifications of TaCYP81D5 and the control gene TaSRO1. Figure S4 Subcellular localization of the TaCYP81D5‐GFP fusion protein. Figure S5 TaCYP81D5 contributes to the salinity tolerance of A. thaliana. Figure S6 The effect on salinity tolerance of mutagenizing CYP81Dx. Figure S7 The contribution of CYP81D genes to salinity tolerance in additional genetic materials. Figure S8 The involvement of TaCYP81D5 in H2O2 tolerance and ROS scavenging in Arabidopsis. Figure S9 Evidence of the involvement of Zat12 in the contribution made by TaCYP81D5 to salinity tolerance in Arabidopsis. [file PBI-18-791-s002.docx]

**Supplemental Figure**


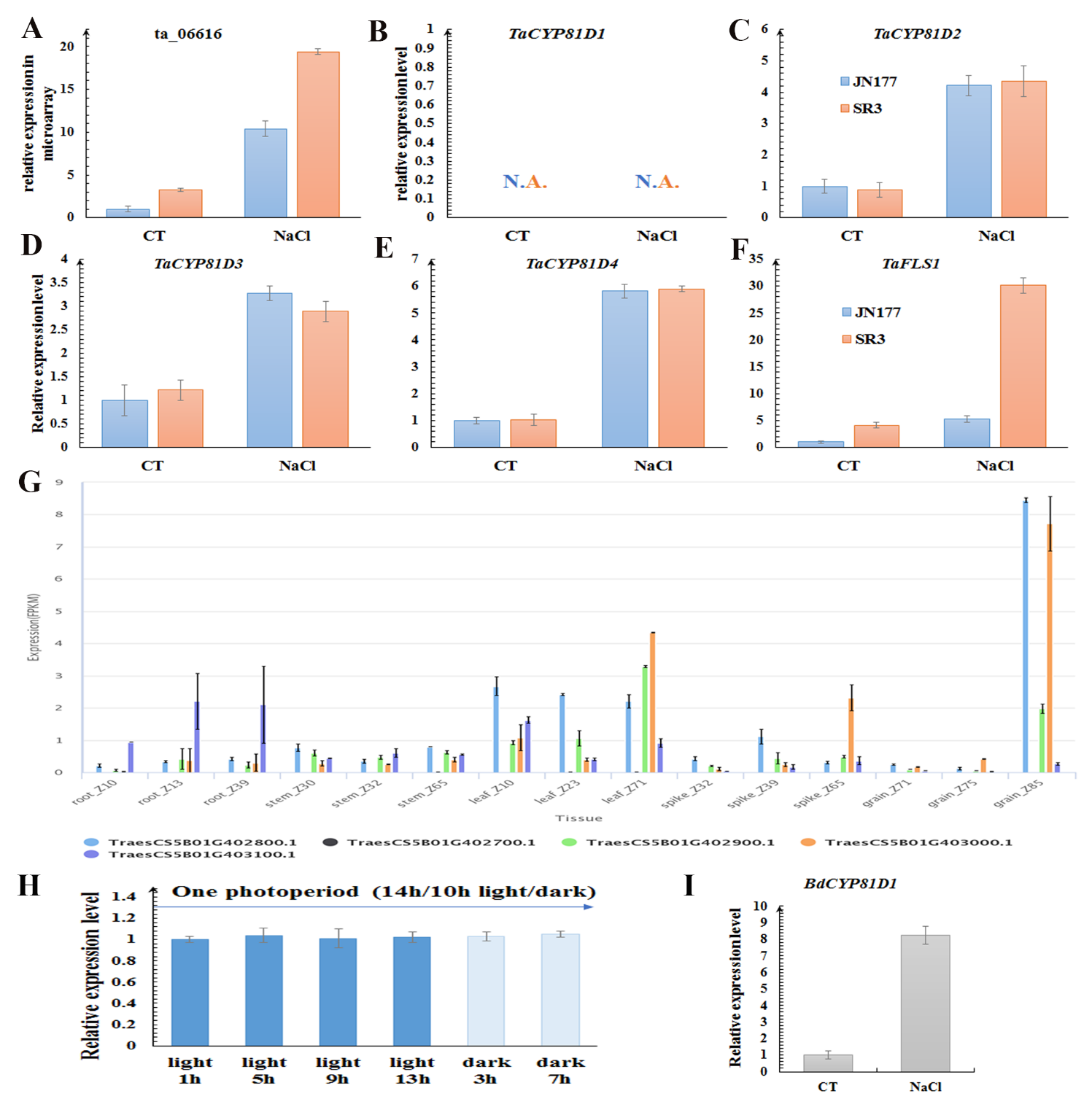


**Fig. S1. The transcriptional profiles of *CYP81Dx* genes.** (A) cDNA microarray assay of ta_06616 transcription in cv SR3 and JN177 as affected by exposure to salinity stress. (B)-(F) the expression patterns of *TaCYP81D1*, *TaCYP81D2*, *TaCYP81D3*, *TaCYP81D4*, and *TaFLS1* (positive control, Wang et al., 2014). *TaEF1-α* (M90077) (Paolacci et al., 2009) was chosen as the endogenous control. (G) The spatial expression of *TaCYP81D1*, *TaCYP81D2*, *TaCYP81D3*, *TaCYP81D4*, and *TaCYP81D5* in cv Chinese Spring (data collected from the RNA-seq libraries of Choulet et al. 2014). (H) The photoperiod effect on *TaCYP81D5* expression. *TaEF1-α* (M90077) (Paolacci et al., 2009) was chosen as the endogenous control. (I) The salinity inducible expression of *BdCYP81D1* in *Brachypodium distachyon*. *BdACTIN* (DV478555) was chosen as the endogenous control. Each bar represents the mean ± SD of at least three biological replicates. N.A.: no data available.


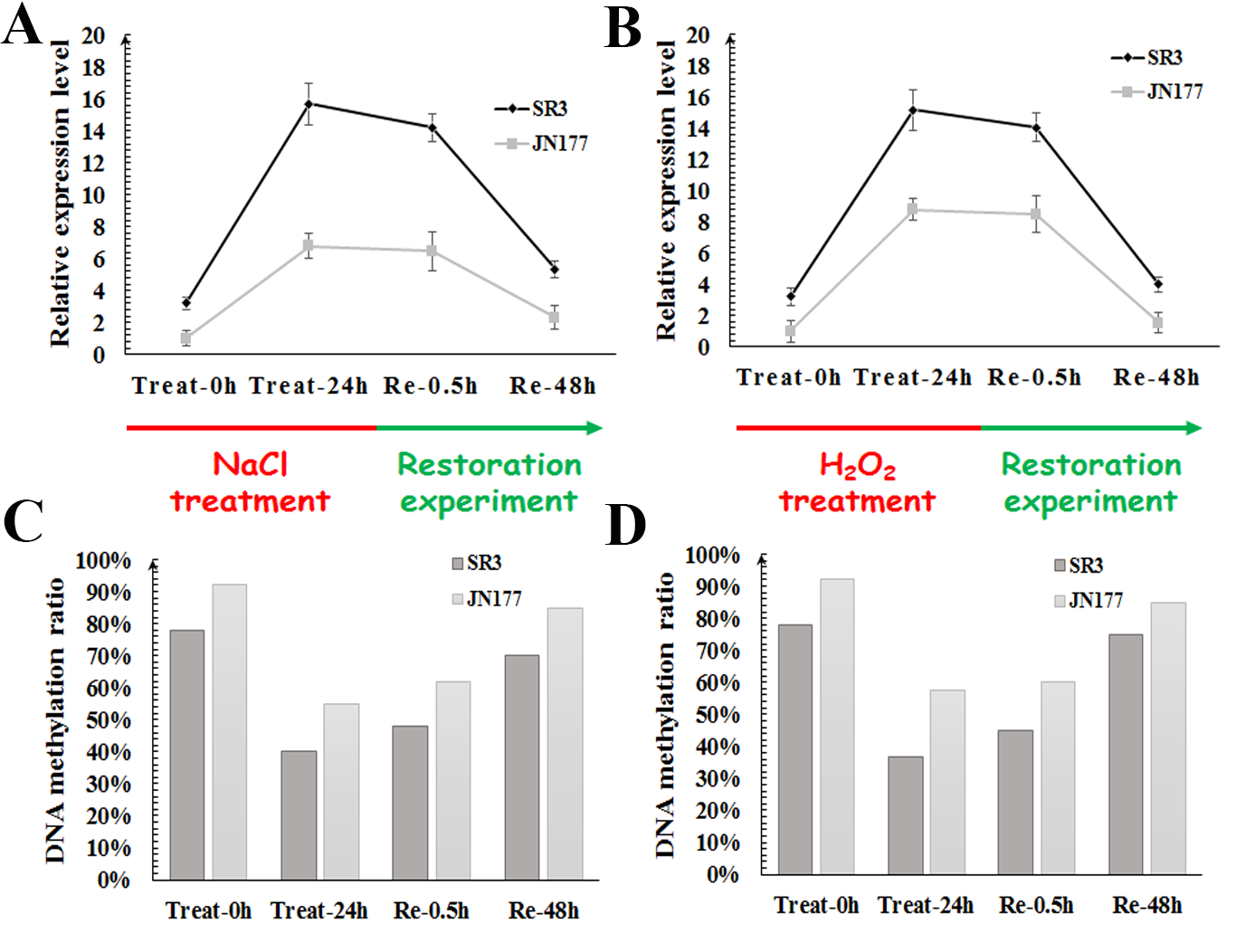


**Fig. S2. The transcriptional abundance (A, B) and DNA methylation ration (C, D) of *TaCYP81D5* in response to abiotic stress (salinity stress, A and C; H_2_O_2_ stress, B and D) and the subsequently restoring treatment.** *TaEF1-α* (M90077) (Paolacci et al., 2009) was chosen as the endogenous control for relative expression level measurement. Each bar represents the mean ± SD of at least three biological replicates.


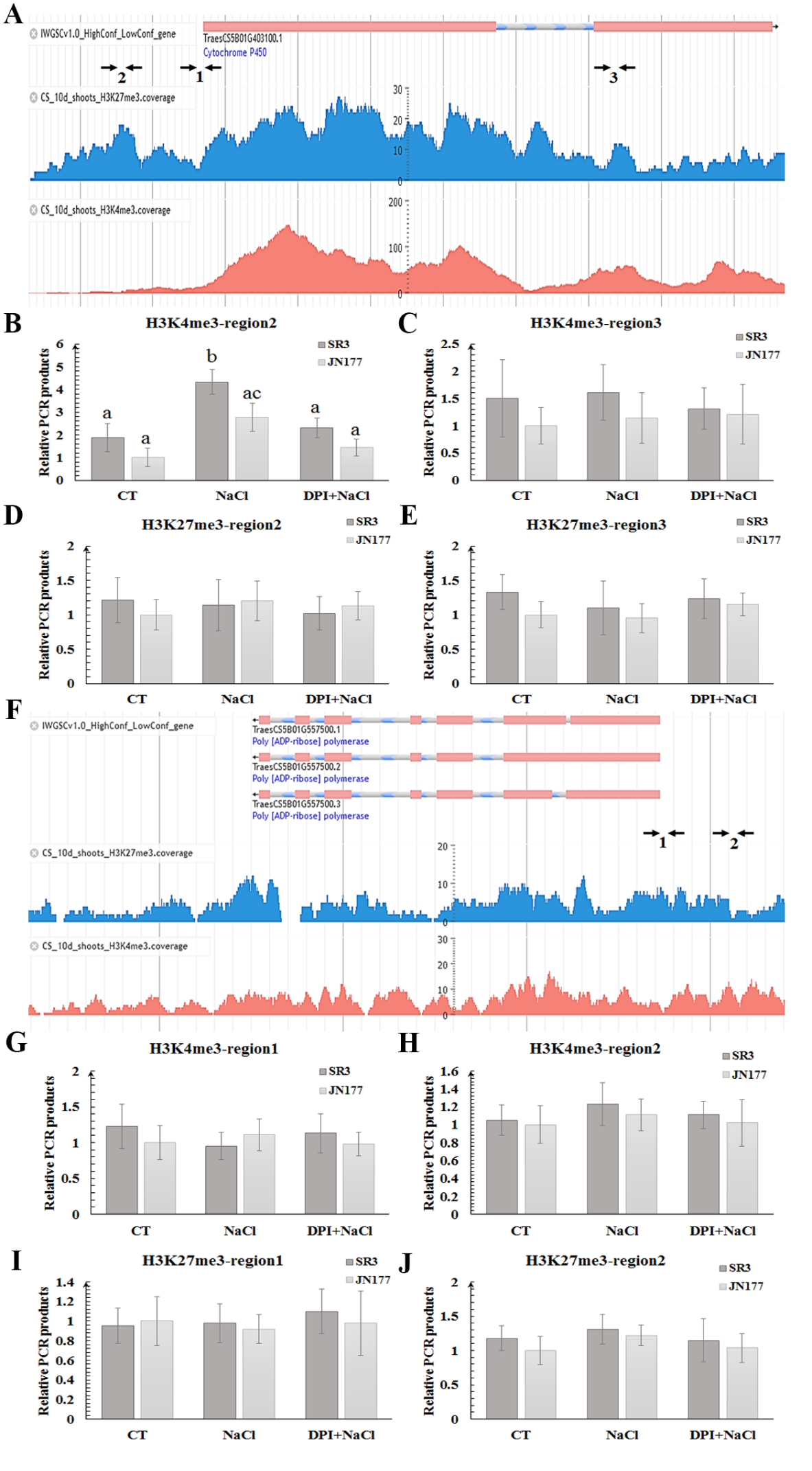


**Fig. S3. Histone modifications of *TaCYP81D5* and the control gene *TaSRO1***. The histone modifications (H3K27me3 and H3K4me3) of (A) *TaCYP81D5* and (F) *TaSRO1* according to the ChIP-seq data (Ramírez-González et al., 2018 ) (which is available on the Triticeae Multi-omics Center: <http://202.194.139.32/>). Arrows and numbers indicate the designed region for ChIP-qPCR. (B, C) The H3K4me3 level and (D, E) the H3K27me3 level of *TaCYP81D5* within (B, D) region 2 and (C, E) region 3. (G, H) The H3K4me3 level and (I, J) the H3K27me3 level of *TaCYP81D5* within (G, I) region 1 and (H, J) region 2. Each bar represents the mean ± SD of at least three biological replicates. Different letters on the top of the bars indicate significance using the one-way Waller-Duncan test (*P* < 0.05).


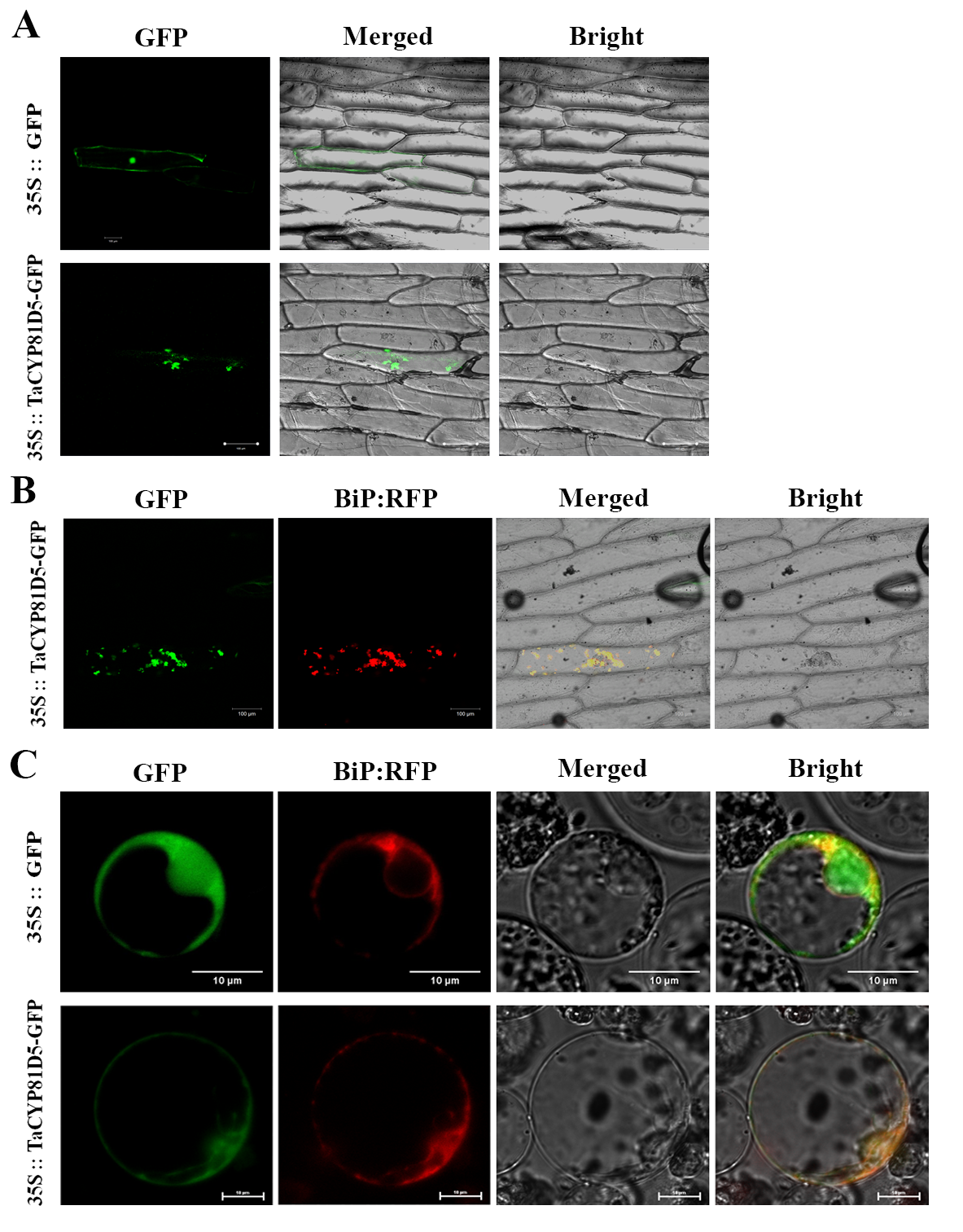


**Fig. S4. Subcellular localization of the TaCYP81D5-GFP fusion protein.** (A) The control GFP and fused TaCYP81D5-GFP in onion epidermal cells. Bar: 100 μm. (B) The ER colocalization of TaCYP81D5-GFP and BiP:RFP (a subcellular marker of ER) in onion epidermal cells. Bar: 100 μm. (C) Subcellular localization of TaCYP81D5-GFP fusion protein in wheat protoplast. Bar: 10μm.


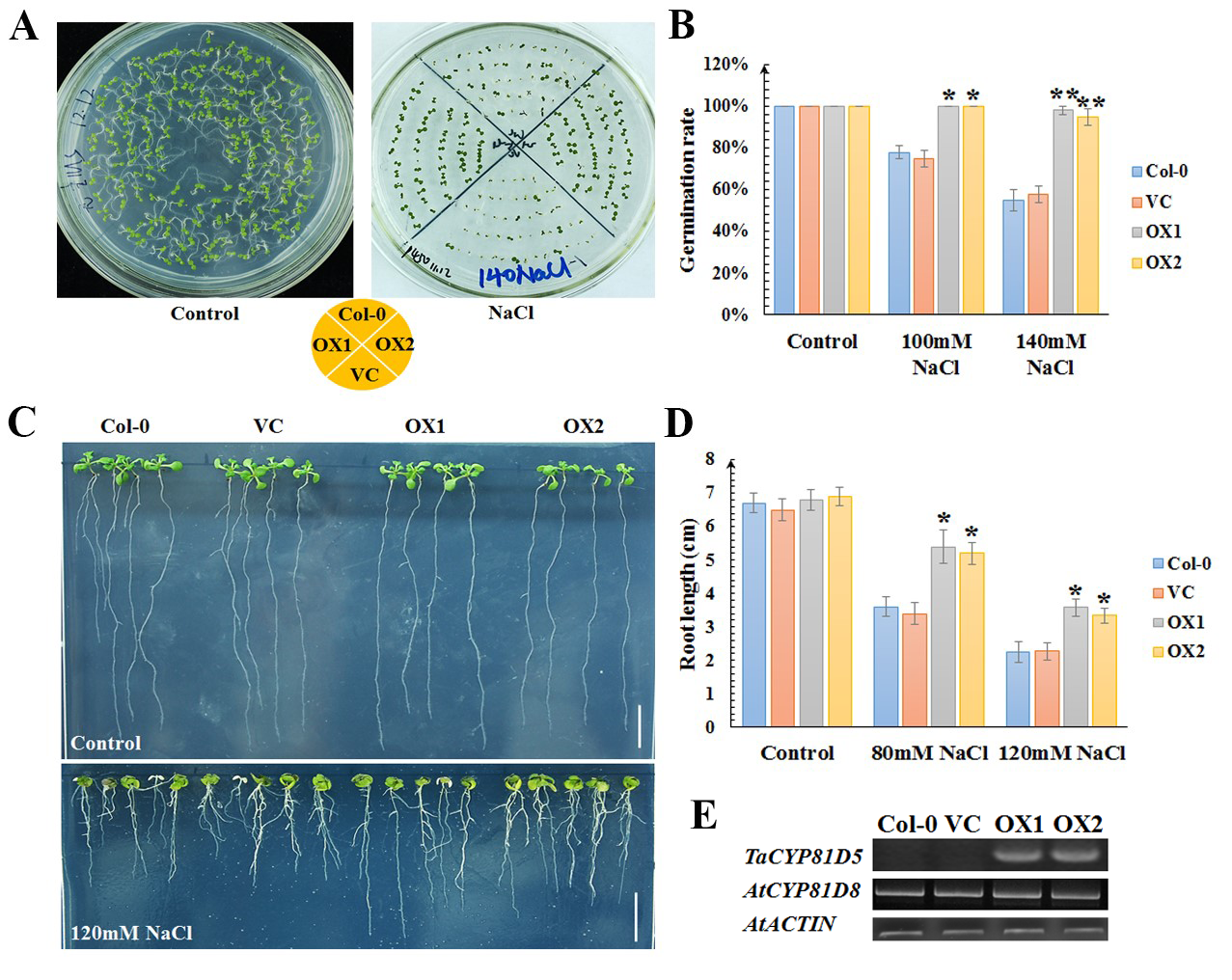


**Fig. S5. *TaCYP81D5* contributes to the salinity tolerance of *A*. *thaliana*.** (A) The germination rate and (B) the development of green cotyledons in Arabidopsis wild-type Col-0, VC (transgenic line carrying an empty pSTART vector) and OX1 and OX2 (transgenic lines heterologously expressing *TaCYP81D5*) under control condition or salt stress. (C) The root length and (D) seedling phenotype of Col-0, VC, OX1 and OX2 under control condition or salt stress. (E) RT-PCR analysis indicated that the *TaCYP81D5* transgene was successfully transcribed in Arabidopsis. Data are presented as mean ± SE of at least three biological replicates. Columns marked with one asterisk indicate significant differences (*P* < 0.05) using Student’s t test, and double asterisks indicate *P* < 0.01. Bar: 1cm.


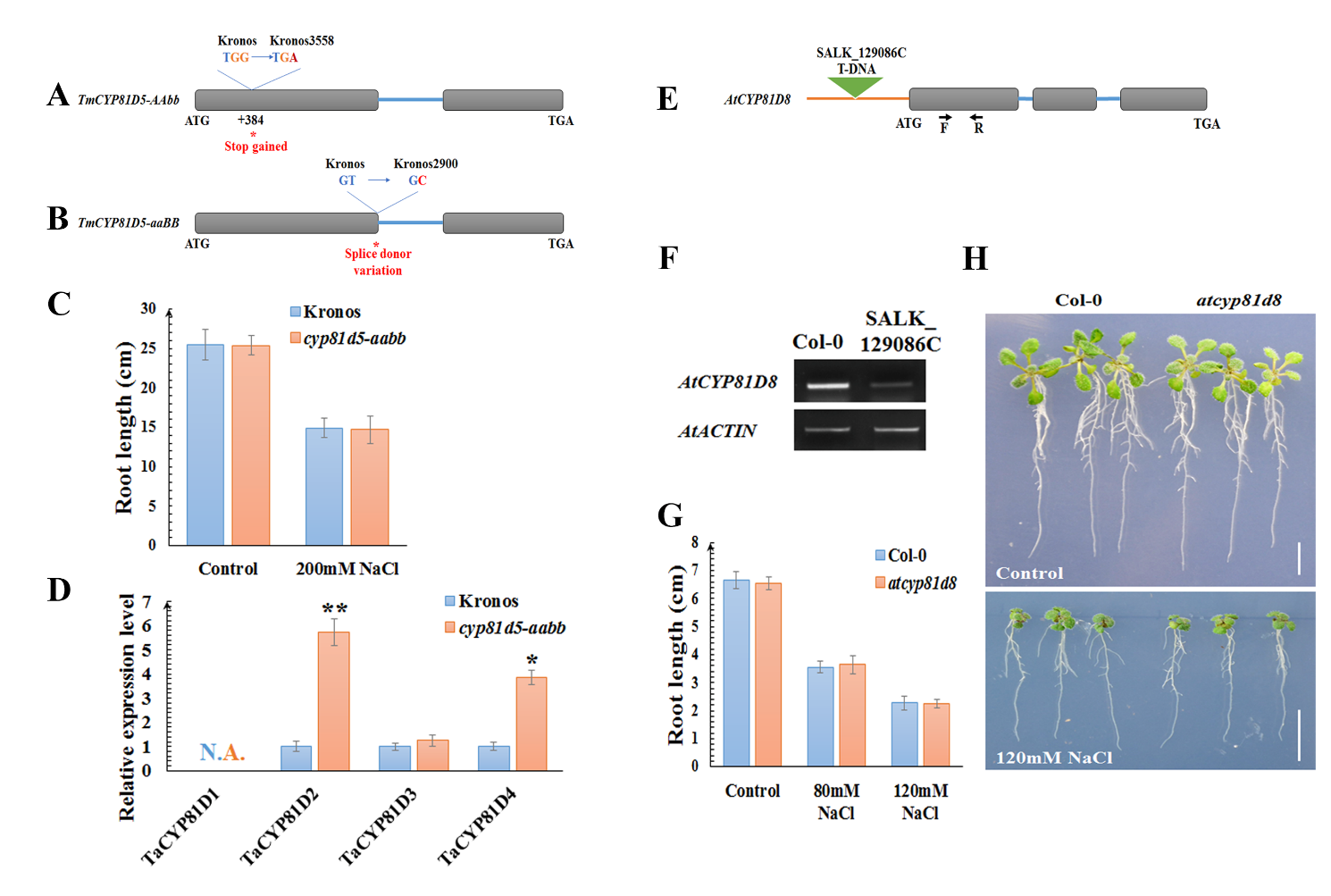


**Fig. S6. The effect on salinity tolerance of mutagenizing *CYP81Dx*.** (A, B) Mutagenesis of *CYP81D5* in wheat. (C) Root growth of wheat cultivar Kronos and its *CYP81D5* mutant (*cyp81d5-aabb*) in the absence and presence of salinity. (D) The abundance of *TaCYP81D1*, *D2*, *D3*, and *D4* transcript in the roots of WT and *cyp81d5-aabb*. *TaEF1-α* (M90077) (Paolacci et al., 2009) was chosen as the endogenous control. Relative transcript abundance of every gene in Kronos was calculated by giving the value 1. (E, F) The T-DNA insertion in the promoter region caused a knock-down of *AtCYP81D8* in Arabidopsis. (G) The root growth and (H) the seedling phenotype of WT *A*. *thaliana* and the loss-of-function *atcyp81d8* mutant. Data are presented as mean ± SE of at least three biological replicates. Columns marked with one asterisk indicate significant differences (*P* < 0.05) using Student’s t test, and double asterisks indicate *P* < 0.01. Bar: 1cm.


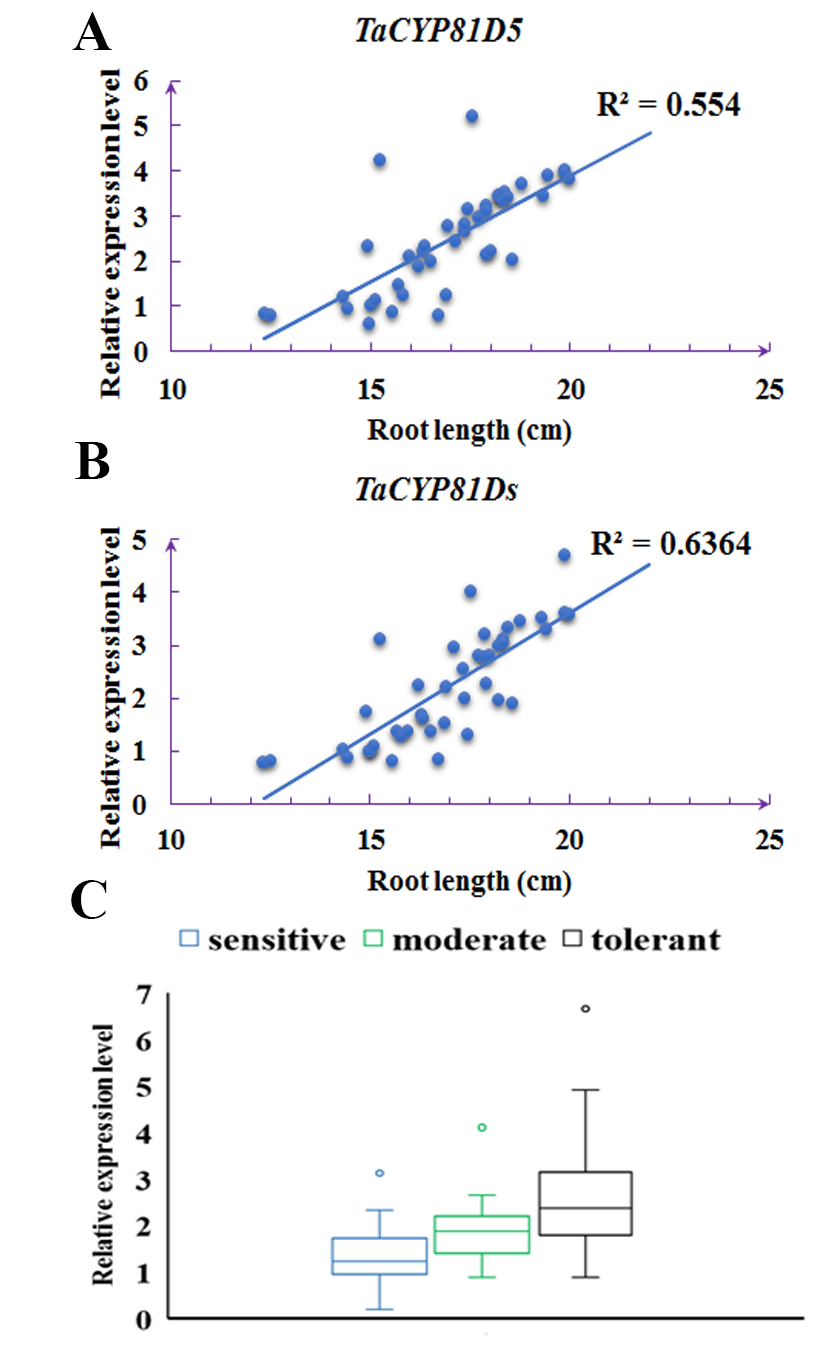


**Fig. S7. The contribution of *CYP81D* genes to salinity tolerance in additional genetic materials.** (A) The association of *TaCYP81D5* expression with root length under salinity stress using the F_2_ seeds of a cross between SR3 and JN177. (B) The association of *TaCYP81Ds* cluster expression with root length under salinity stress using the F_2_ seeds of a cross between cv. SR3 and cv. JN177. (C) The relative expression levels of *TaCYP81Ds* cluster in 20 salinity tolerant, 20 moderate tolerant and 20 sensitive bread wheat accessions. *TaEF1-α* (M90077) (Paolacci et al., 2009) was chosen as the endogenous control. Gene expression level in cv. JN177 was calculated by giving the value 1.


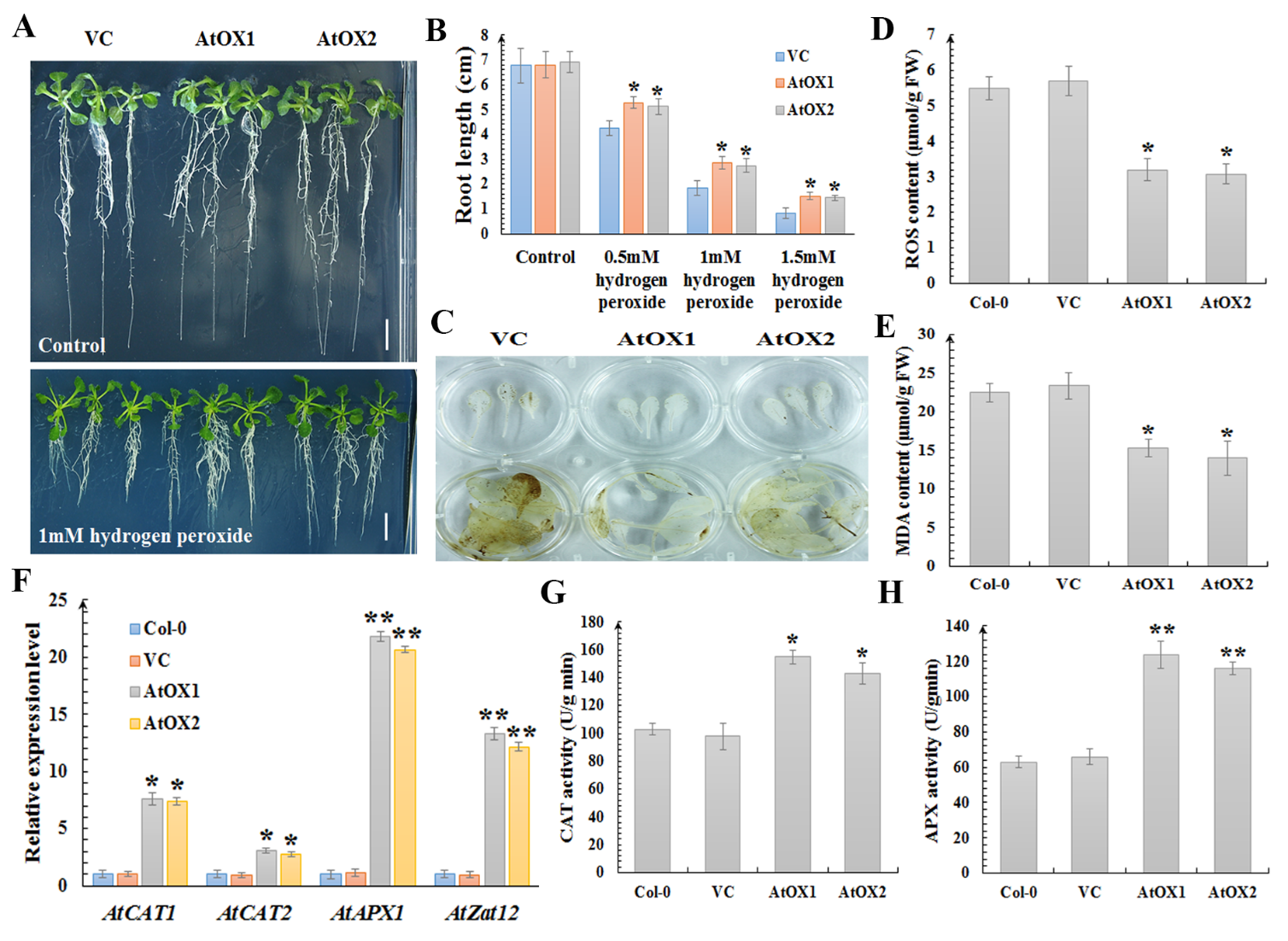


**Fig. S8. The involvement of *TaCYP81D5* in H_2_O_2_ tolerance and ROS scavenging in Arabidopsis.** (A) and (B) The seedling phenotype and root length of VC (Arabidopsis transgenic line carrying an empty pSTART vector), OX1 and OX2 (transgenic lines heterologously expressing *TaCYP81D5*) under control condition or H_2_O_2_ treatment. Bar = 1cm. (C) ROS levels of VC, OX1 and OX2 lines by DAB staining. (D) The H_2_O_2_ and (E) MDA contents in Arabidopsis Col-0, VC, OX1 and OX2 lines. (F) The expression levels of *AtCAT1*, *AtCAT2*, and *AtAPX1* in roots of Col-0, VC, OX1 and OX2 lines. *AtActin2* (At3g18780) was the constitutive normalization control. Relative transcript abundance of every gene in Col-0 was calculated by giving the value 1. (G) CAT and (H) APX activities of Arabidopsis Col-0, VC, OX1 and OX2 lines. Each bar of the columns represents the mean ± SE of at least three biological replicates. Columns marked with one asterisk indicate significant differences (*P* < 0.05) using Student’s t test, and double asterisks indicate *P* < 0.01.


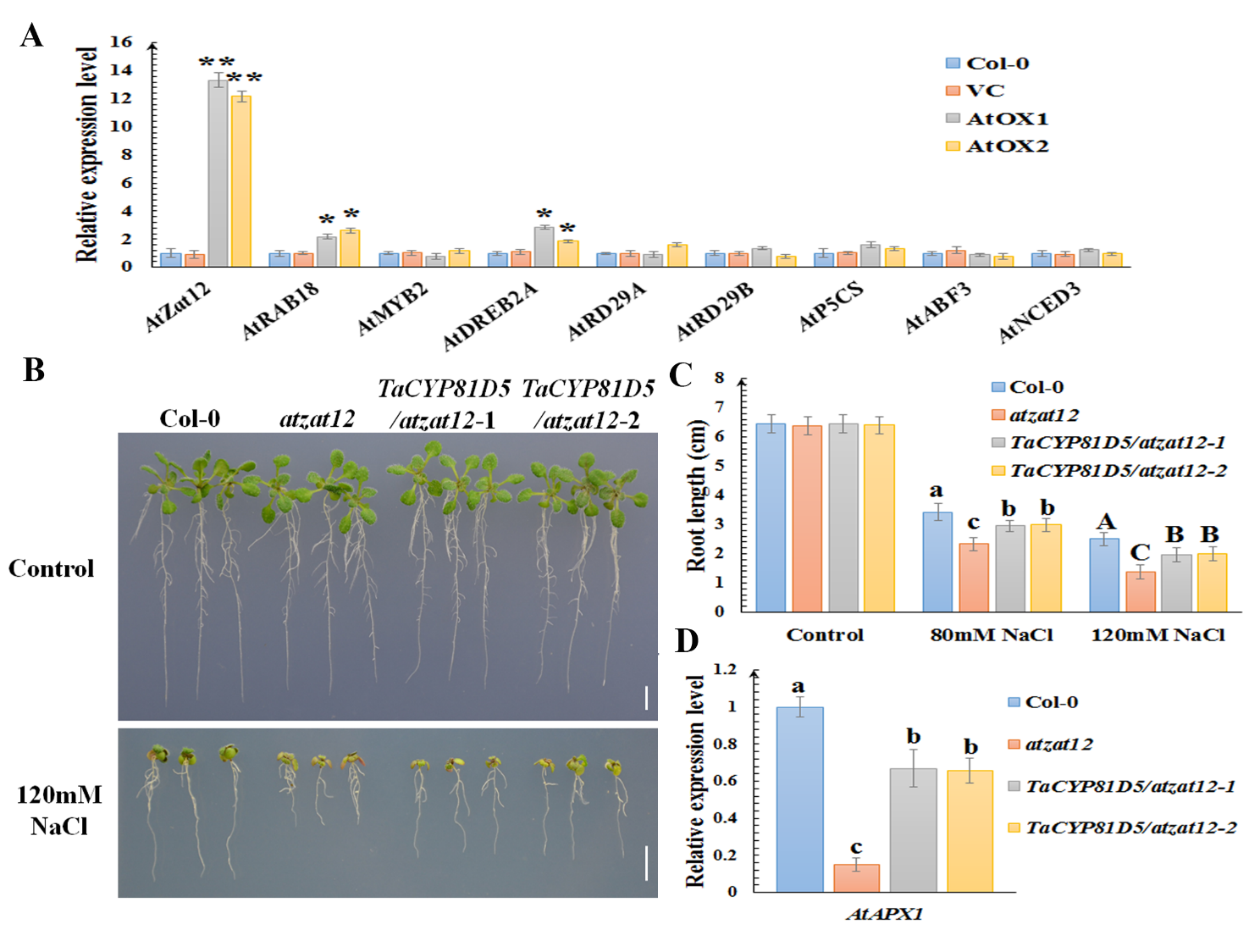


**Fig. S9. Evidence of the involvement of Zat12 in the contribution made by TaCYP81D5 to salinity tolerance in Arabidopsis.** (A) Relative transcription levels in 2-week-old seedlings of marker genes involved in stress-responsive signaling pathways. Columns marked with one asterisk indicate significant differences (*P* < 0.05) using Student’s t test, and double asterisks indicate *P* < 0.01. (B) The seedling phenotype and (C) root length of Arabidopsis wild-type Col-0, *atzat12* (SALK_037357), and *TaCYP81D5*-OX/*atzat12*-1 and *TaCYP81D5*-OX/*atzat12*-2 (a constitutive expressor of *TaCYP81D5* in the background of the *atzat12* mutant) both in the absence and presence of salinity stress. Bar: 1 cm. (D) The abundance of *AtAPX1* transcript in roots of Col-0, *atzat12*, *TaCYP81D5*-OX/*atzat12*-1, and *TaCYP81D5*-OX/*atzat12*-2 lines. *AtActin2* (At3g18780) was the constitutive normalization control. Relative transcript abundance of every gene in Col-0 was calculated by giving the value 1. Each bar represents the mean ± SD of at least three biological replicates. Different letters on the top of the bars indicate significance using the one-way Waller-Duncan test (*P* < 0.05).
