# Supplementary material for: TaCYP81D5, one member in a wheat cytochrome P450 gene cluster, confers salinity tolerance via reactive oxygen species scavenging
Source: Plant Biotechnol J. 2019 Sep 17;18(3):791–804. doi: 10.1111/pbi.13247 (PMC7004906; doi:10.1111/pbi.13247)
Supplement: Supplementary file 2 — Table S1 List of genes used in this study Table S2 Primers used in this study [file PBI-18-791-s001.docx]

**Table S1. List of genes used in this study**

| Species | Symbol | Gene ID in NCBI/ Phytozome |
| --- | --- | --- |
| Bread wheat (*Triticum aestivum*) | *TaCYP81D1* | TraesCS5B01G402700.1 |
|  | *TaCYP81D2* | TraesCS5B01G402800.1 |
|  | *TaCYP81D3* | TraesCS5B01G402900.1 |
|  | *TaCYP81D4* | TraesCS5B01G403000.1 |
|  | *TaCYP81D5* | TraesCS5B01G403100.1 |
|  | *TaFLS1* | KJ193852 |
|  | *TaEF1-α* | M90077 |
|  | *TaAPX* | TC369354 |
|  | *TaCAT* | AJ007349 |
|  | *TaNOX* | AY561153 |
|  | *TaAOX* | TraesCS2A01G439400.1 |
|  | *TaZat12* | TraesCS1B01G053200.1 |
|  | *TaZFP36* | TraesCS7A01G160700.2 |
|  | *TaSRO1* | TraesCS5B01G557500.2 |
| *Arabidopsis thaliana* | *AtCYP81D5* | At4g37320 |
|  | *AtCYP81D4* | At4g37330 |
|  | *AtCYP81D3* | At4g37340 |
|  | *AtCYP81D2* | At4g37360 |
|  | *AtCYP81D8* | At4g37370 |
|  | *AtAPX1* | At1g07890 |
|  | *AtCAT1* | At1g20630 |
|  | *AtCAT2* | At4g35090 |
|  | *AtZAT12* | At5g59820 |
|  | *AtACTIN2* | At3g18780 |
| *Brachypodium distachyon* | *BdCYP81D1* | Bd1g07930 |
|  | *BdACTIN* | DV478555 |
| Rice (*Oryza sativa*) | *OsCYP81D1* | Os03g55230 |
|  | *OsCYP81D2* | Os03g55240 |
|  | *OsCYP81D3* | Os03g55250 |
|  | *OsCYP81D4* | Os03g55260 |
|  | *OsZat12* | Os05g0114400 |
|  | *OsZFP36* | Os03g0437200 |
| Barley (*Hordeum vulgare*) | *HvCYP81D1* | Hv5Hr1G096930 |
|  | *HvCYP81D2* | Hv5Hr1G096940 |
|  | *HvCYP81D3* | Hv5Hr1G096950 |
|  | *HvCYP81D4* | Hv5Hr1G096970 |
| *Sorghum vulgare* | *SbCYP81D1* | Sb01g082200 |
|  | *SbCYP81D2* | Sb01g082300 |
|  | *SbCYP81D3* | Sb01g082400 |
|  | *SbCYP81D4* | Sb01g082500 |

**Table S2. Primers used in this study**

| N.o | Sequence | Purpose |
| --- | --- | --- |
| TaCYP81D5-F | ATGGATAAGGCATACATTGC | cDNA, gDNA and promoter cloning. |
| TaCYP81D5-R | TCAGAGGCTCTCAAGCACGT |  |
| TaZat12-F | ATGATGAAGAGGTTTGCTTTCG |  |
| TaZat12-R | CTAGACGCAGTCCACCGCCAT |  |
| TaAPX-P-F | GGCTGGGCCGGGAACA |  |
| TaAPX-P-R | GCGAGGCGGAGCATGAG |  |
| TaAPX-mP-F | CCCCTGGCCATCGCTCGCGT |  |
| TaAPX-mP-R | CACGCGAGCGATGGCCAGGGG |  |
| TaCYP81D5-221F | CGCGGATCCATGGATAAGGCATACATTGC | *35S*::*TaCYP81D5*-*GFP* construction for subcellular localization. |
| TaCYP81D5-221R | CCCAAGCTTGAGGCTCTCAAGCACGTC |  |
| TaCYP81D5-OE-F | CCCAAGCTTATGGATAAGGCATACATTGC | *Ubi*::*TaCYP81D5* construction for wheat transformation. |
| TaCYP81D5-OE-R | CGCGGATCCTCAGAGGCTCTCAAGCACGTC |  |
| TaCYP81Ds-RNAi-SF | CGCGGATCCATGGATAAGGCATACATTGC | Construction for wheat RNAi generation. |
| TaCYP81Ds-RNAi-SR | CGGGGTACC GAGAGCGCGATGCCGT |  |
| TaCYP81Ds- RNAi-AF | CGAGCTCATGGATAAGGCATACATTGC |  |
| TaCYP81Ds- RNAi-AR | GACTAGTGAGAGCGCGATGCCGT |  |
| TaCYP81D5-AtF | CCCAAGCTTATGGATAAGGCATACATTGC | *pSTART*::*TaCYP81D5* construction for Arabidopsis transformation. |
| TaCYP81D5-AtR | CGCGGATCCTCAGAGGCTCTCAAGCACGTC |  |
| *Atcyp81d8*-LP | CCGAGAAAGACGAAACACAAG | Phenotyping for Arabidopsis mutants. |
| *Atcyp81d8*-RP | GAGAGACTCGCGATTGCATAC |  |
| *Atzat12*-LP | CAGTGTCCAATTCTCTCTGGC |  |
| *Atzat12*-RP | GCTCCATCTCTGATTGATTCG |  |
| LBb1.3 | ATTTTGCCGATTTCGGAAC |  |
| TaCYP81D5-mF1 | TTTTAGGTAAGGTTATTAATGGTAGGTATA | Bisulfate PCR. |
| TaCYP81D5-mR1 | ACTTCTCCAAAAAATAAAAATAACC |  |
| TaCYP81D5-mF2 | TTTAAGATTTTAAGATGAAATTAGAATATT |  |
| TaCYP81D5-mR2 | TCTACACATAATCCATTAAAAAAAC |  |
| TaWRKY40-McrF | CCCTGCAGGGCCAGTTCAC | McrBC-qPCR. |
| TaWRKY40-McrR | CGCCGCGTACGCGAGGCCG |  |
| TaCYP81D5-McrF1 | ATTTCCAACCGTGACTTCG |  |
| TaCYP81D5-McrR1 | GGAACTCCCAAGGACTACAAT |  |
| TaCYP81D5-McrF2 | TGTTTGGGCAGACGATGAT |  |
| TaCYP81D5-McrR2 | AGTGGGATTTGTTCCTTCG |  |
| TaCYP81D5-HM-F1 | CTTTCAACATTTCCACGAT | H3K4me3 and H3K27me3 CHIP-qPCR. |
| TaCYP81D5-HM-R1 | ATTGCGTGGTTCATCTTC |  |
| TaCYP81D5-HM-F2 | TTTCCAACCGTGACTTCG |  |
| TaCYP81D5-HM-R2 | ACAGGGCATCTTTCATCT |  |
| TaCYP81D5-HM-F3 | GATCTCACCAATAACCCTCGTT |  |
| TaCYP81D5-HM-R3 | GGTTCAGCAGCAGCGACAT |  |
| TaSRO1-HM-F1 | CTTGTGGATAACATGGCTCG |  |
| TaSRO1-HM-R1 | ATTTTACACAAAGCTCAACTAAAAC |  |
| TaSRO1-HM-F2 | GCAACTTACTACTGCCAATG |  |
| TaSRO1-HM-R2 | CAGTAAGCCTTTCAACTA |  |
| TaCYP81D5-qF | GCCTTCATGCTGCGTCTCA | qRT/RT-PCR in wheat. |
| TaCYP81D5-qR | GTTCCAGCCCCGAATAAAT |  |
| TaCYP81D1-qF | GGATGCTGGGCTTGGTTCTGG |  |
| TaCYP81D1-qR | TCAACCTCAGCATCACCAACCCT |  |
| TaCYP81D2-qF | CAACGCCGTGGTGGTGTCCT |  |
| TaCYP81D2-qR | CCGCCGTAGGAGACGAGCAT |  |
| TaCYP81D3-qF | GGGATTACCTGCCCGTGTTGC |  |
| TaCYP81D3-qR | CGGCGGCCAGGATCTTGTTC |  |
| TaCYP81D4-qF | TGCTCCTCAATCTCCAGAAGACG |  |
| TaCYP81D4-qR | AGCAGCGACATCGCCCACTC |  |
| TaCYP81Ds-qF | GCTCATGGAGACCATCGC |  |
| TaCYP81Ds-qR | ACACGTCGAACCACCGCA |  |
| TaFLS1-qF | GCAGGAGGAGAAGCAGCGGT |  |
| TaFLS1-qR | GCGACGTTGTGGAAGAGGAAGT |  |
| TaEF-α-qF | GGTTAAGATGATTCCCACCAAGCC |  |
| TaEF-α-qR | GACAACACCAACAGCAACAGTCTG |  |
| TaCAT-qF | CAAGGGCTTCTTCGAGGTCAC |  |
| TaCAT-qR | TGTAGAAGGTCCACTCCGGGTAG |  |
| TaAPX-qF | GGTTTGAGTGACCAGGACATTG |  |
| TaAPX-qR | GCATCCTCATCCGCAGCAT |  |
| TaNOX-qF | ATGTTCGGCAACTTGGTGACT |  |
| TaNOX-qR | CGTCTGCTCTAAGAAGACCACTTTT |  |
| TaZat12-qF | GATGCTCCGTCTGCGGTCTT |  |
| TaZat12-qR | GCAAGGTGGGTGGTTCAGGTC |  |
| TaZFP36-qF | CGACTACCTCGCCATCTGCC |  |
| TaZFP36-qR | CTTGCGGTGGCTCGACTTG |  |
| BdCYP81D1-qF | GGACGAGATCATCCCGCACCT | qRT/RT-PCR in *Brachypodium distachyon* |
| BdCYP81D1-qR | TCCTTCGCTCCGCTTCAATCA |  |
| BdACTIN-qF | CCTGAAGTCCTTTTCCAGCC |  |
| BdACTIN-qR | AGGGCAGTGATCTCCTTGCT |  |
| AtCYP81D8-qF | TCTCAATCCCTAAACAATGCTCC | qRT/RT-PCR in Arabidopsis. |
| AtCYP81D8-qR | AAGTTTGGTCTGTTCGCCAGTA |  |
| AtActin2-qF | TATGAATTACCCGATGGGCAAG |  |
| AtActin2-qR | TGGAACAAGACTTCTGGGCAT |  |
| AtAPX1-qF | GTCCATTCGGAACAATGAGGTTTGAC |  |
| AtAPX1-qR | GTGGGCACCAGATAAAGCGACAAT |  |
| AtCAT1-qF | CGCCGATTTGCGAGATACACACAG |  |
| AtCAT1-qR | GACCTCGAGTTCCGACAGTCAAAGA |  |
| AtCAT2-qF | TCCCGTCGAGGTATGACCAGGTT |  |
| AtCAT2-qR | CTTGCCAGCTTCTGTCCCAAAGACT |  |
| AtZat12-qF | GGCGAATTGTTTGATGCTTT |  |
| AtZat12-qR | CAAGCCACTCTCTTCCCACT |  |
| AtAAO3-qF | TCATTCAACAAGCGTATGGTCAG |  |
| AtAAO3-qR | GGTATTTTGTAGTCCCAAGTGCCT |  |
| AtNCED3-qF | CAATCATCAAACTCTCCCGCC |  |
| AtNCED3-qR | TCTCGTGGCTGACAAGGAAAC |  |
| AtP5CS-qF | CTTGTGATACGGATATGGCAAAGCG |  |
| AtP5CS-qR | CCTTGGTCCACCATACAAAGTGACTCC |  |
| AtRAB18-qF | GGAGAAGTTGCCAGGTCATC |  |
| AtRAB18-qR | ACCGGGAAGCTTTTCCTTGATC |  |
| AtRD29A-qF | CTTGATGGTCAACGGAAGGT |  |
| AtRD29A-qR | CAATCTCCGGTACTCCTCCA |  |
| AtRD29B-qF | AGAAGGAATGGTGGGGAAAG |  |
| AtRD29B-qR | CAACTCACTTCCACCGGAAT |  |
| AtMYB2-qF | AACGTCTTCGAATTCTCCGGCTGA |  |
| AtMYB2-qR | ATCGTTGAACTCTCCGAAACCCGT |  |
| AtABF3-qF | AACGCTGGGAGAGATGACTTTGGA |  |
| AtABF3-qR | TCCCAAGACCTCCATTACTGCCAA |  |
| AtDREB2A-qF | AAACCTGTCAGCAACAACAGCAGG |  |
| AtDREB2A-qR | TTAAGCCTGCAAACACATCGTCGC |  |
